# Moxibustion and the gut microbiota–inflammation axis: a systematic review and meta-analysis of clinical and preclinical studies

**DOI:** 10.3389/fcimb.2026.1890021

**Published:** 2026-10-05

**Authors:** Xiaotao Zhang, Yueyue Guo, Lu Chen, Nian Song, Yang Yang, Jie Kong, Huan Xu, Yulu Liu, Ying Li

**Affiliations:** Jiangsu Province Hospital of Chinese Medicine, Department of Nursing, Affiliated Hospital of Nanjing University of Chinese Medicine, Nanjing, China

**Keywords:** gut microbiota, inflammation, meta-analysis, microbiota–host interaction, moxibustion, systematic review

## Abstract

**Background:**

Gut dysbiosis and aberrant inflammatory signaling are increasingly implicated in the pathogenesis of intestinal and extra-intestinal disorders. Moxibustion has been reported to modulate gut microbiota composition and inflammatory responses, but its overall effects remain incompletely characterized. Objective: To evaluate the effects of moxibustion on gut microbiota-related outcomes and inflammatory mediators in clinical and preclinical studies.

**Methods:**

PubMed, Web of Science, the Cochrane Library, CNKI, WanFang Data, and VIP were searched from database inception to December 31, 2024. Clinical and preclinical studies investigating moxibustion and reporting at least one gut microbiota-related outcome were included. Clinical and preclinical evidence was synthesized separately, with quantitative meta-analysis restricted to eligible preclinical animal outcomes. Microbiota outcomes were pooled using ratios of means (ROMs), whereas inflammatory mediators were synthesized using standardized mean differences (SMDs) under random-effects models. Metabolite- and pathway-related findings were narratively synthesized.

**Results:**

Thirty-one reports representing 30 unique studies were included, comprising 25 preclinical animal studies (26 reports) and 5 clinical studies. In animal studies, moxibustion increased Chao1 (ROM = 1.079, 95% CI [1.034–1.127]; P = 0.0005), ACE (ROM = 1.100, 95% CI [1.044–1.160]; P = 0.0004), and Shannon diversity (ROM = 1.047, 95% CI [1.015–1.080]; P = 0.0037), whereas Simpson diversity was not significantly altered. No significant pooled effects were detected for the major phyla. *Ruminococcus* decreased (ROM = 0.643, 95% CI [0.426–0.972]; P = 0.0364), whereas the other taxa retained in the main analysis showed no statistically significant pooled effects. Moxibustion reduced TNF-α (SMD = −1.648, 95% CI [−2.659 to −0.637]; P = 0.0014) and IL-1β (SMD = −1.861, 95% CI [−2.419 to −1.303]; P < 0.0001), whereas IL-6 and IL-10 were not significantly altered. Clinical findings were sparse and directionally inconsistent. Certainty of evidence for the evaluable prespecified primary microbiota outcomes was very low. Three studies further suggested potential modulation of short-chain fatty acids, amino acid metabolism, and microbial functional pathways.

**Conclusion:**

Preclinical evidence indicates that moxibustion is associated with increased microbial richness and diversity and reduced selected pro-inflammatory mediators, while taxon-specific responses are highly heterogeneous and clinical confirmation remains insufficient.

**Systematic review registration:**

https://www.crd.york.ac.uk/PROSPERO/, identifier CRD42025639714.

## 1 Introduction

The gut microbiota is a dynamic host-associated ecosystem that plays essential roles in epithelial barrier maintenance, immune regulation, metabolic homeostasis, and neuroimmune communication (Fan and Pedersen, 2021). Through microbial metabolites such as short-chain fatty acids, bile acids, and tryptophan derivatives, gut microbes participate in bidirectional host–microbe crosstalk and influence both intestinal and extraintestinal physiology (Cryan et al., 2019; Zhang et al., 2023). Recent microbiome studies further indicate that microbiota-associated signaling networks are closely linked to mucosal immunity, inflammatory regulation, and epithelial barrier integrity, thereby influencing disease susceptibility and progression across multiple organ systems (Forbes et al., 2016; Zheng et al., 2020). Accumulating evidence from inflammatory bowel disease (IBD) (O'Sullivan et al., 2025; Zheng et al., 2024), irritable bowel syndrome (IBS) (Cheng et al., 2024; Li et al., 2024), obesity (Fan and Pedersen, 2021), rheumatoid arthritis (Zhang et al., 2015), and other chronic inflammatory and neuroimmune disorders (Morais et al., 2021) indicates that dysbiosis is not limited to reduced diversity or altered taxonomic abundance, but is also associated with impaired barrier function, aberrant immune activation, altered microbial metabolism, and persistent low-grade inflammation. Consequently, increasing attention has been directed toward interventions capable of modulating the gut microbiota–inflammation axis.

Moxibustion is a traditional heat-based therapeutic intervention that involves combustion of *Artemisia argyi*-derived moxa at specific body sites or acupoints. Although widely used in East Asian medicine, accumulating evidence suggests that its biological effects may extend beyond local thermal stimulation and involve microbiota-associated immune and metabolic regulation. Experimental studies have shown that moxibustion may alter gut microbial composition, regulate microbial metabolites, improve intestinal barrier function, and attenuate inflammatory responses across multiple disease models. In a colorectal cancer liver metastasis model, moxibustion reduced liver metastatic burden while partially restoring gut microbial dysbiosis (Song et al., 2023). In aged female Wistar rats, mild moxibustion at Guanyuan (RN4) increased *Lactobacillus* abundance and short-chain fatty acid levels and attenuated chronic low-grade inflammation (Ouyang et al., 2022). In osteoarthritis models, moxibustion shifted the gut microbiota toward a healthier ecological profile and reduced inflammation-associated taxa, including *Ruminococcus* and Proteobacteria (Fu et al., 2024). Moxibustion has also been reported to alleviate inflammatory activity in rheumatoid arthritis and ulcerative colitis models, including modulation of pro-inflammatory cytokines and immune balance (Zhong et al., 2020; Zhu et al., 2024). Collectively, these findings support the possibility that moxibustion may exert part of its biological effects through modulation of the microbiota–metabolite–immune network rather than through isolated local stimulation alone.

Interest in microbiota-targeted non-pharmacological interventions has increased substantially in recent years. Alongside dietary modulation, exercise, probiotics, and fecal microbiota transplantation, emerging evidence suggests that physical or neuromodulatory interventions may also influence microbial ecology and host inflammatory responses (Mailing et al., 2019; Morais et al., 2021). Experimental studies further indicate that neuroimmune regulation, vagal signaling, mucosal immunity, and intestinal barrier integrity may contribute to microbiota-associated responses after moxibustion (Bonaz et al., 2018; Peterson and Artis, 2014; Zheng et al., 2020). Moreover, recent studies suggest that moxibustion and acupuncture may share overlapping but non-identical effects on microbial metabolic pathways and inflammatory signaling, highlighting the potential mechanistic specificity of moxibustion-related microbiota modulation (Yang et al., 2024). Evidence synthesis in this field, however, remains limited. Previous reviews have often combined acupuncture and moxibustion as a single intervention category, which may obscure the specific effects of moxibustion (Bae et al., 2024). This distinction is mechanistically relevant because different physical interventions may influence microbial ecology, neuroimmune signaling, and inflammatory regulation through partially overlapping but non-identical pathways. In addition, existing studies vary substantially in disease background, sequencing platform, intervention protocol, and outcome reporting, making interpretation difficult. Importantly, whether moxibustion independently modulates gut microbiota composition and inflammation-related outcomes across clinical and preclinical evidence remains unclear.

Accordingly, this systematic review and meta-analysis aimed to evaluate the independent effects of moxibustion on gut microbiota-related outcomes and inflammatory mediators while preserving the distinction between clinical and preclinical evidence. We quantitatively synthesized preclinical animal data for microbial diversity, major taxonomic groups, and inflammatory mediators, while clinical evidence and metabolite- or pathway-related findings were synthesized separately where quantitative pooling was not methodologically appropriate. We further examined the robustness and heterogeneity of the preclinical findings through risk-of-bias sensitivity analyses, subgroup analyses, treatment-duration meta-regression, small-study-effect assessment, and evaluation of the certainty of evidence.

## 2 Materials and methods

### 2.1 Study design and registration

This systematic review and meta-analysis was conducted and reported in accordance with the PRISMA 2020 statement (Page et al., 2021). The protocol was prospectively registered in PROSPERO (CRD42025639714) and subsequently published (Zhang et al., 2025). Any deviations from the protocol were documented and considered when interpreting the findings.

### 2.2 Search strategy

A comprehensive literature search was conducted in PubMed, Web of Science, the Cochrane Library, China National Knowledge Infrastructure, Wanfang Data, and VIP Database from inception to December 31, 2024. The search strategy combined controlled vocabulary and free-text terms related to moxibustion and gut microbiota. The main search terms included “moxibustion,” “gut microbiota,” “gut microbiome,” “intestinal flora,” and “intestinal microbiota”. The detailed search strategy is provided in **Supplementary Table S1**.

### 2.3 Eligibility criteria

Studies were eligible if they met the following criteria: (1) an original clinical intervention study or animal experiment; (2) an eligible concurrent comparator; (3) the intervention was moxibustion alone or moxibustion combined with the same background treatment as that used in the control group; and (4) at least one quantitative gut microbiota-related outcome was reported.

Studies were excluded if they met any of the following criteria: (1) reviews, case reports, conference abstracts, comments, protocols, or other non-primary publications; (2) studies in which moxibustion was combined with acupuncture or other interventions and the independent effect of moxibustion could not be isolated; (3) studies with an ineligible comparator; (4) duplicate or overlapping reports that did not provide additional non-overlapping outcome data; or (5) *in vitro* studies.

No restrictions were imposed on disease type, animal species, sequencing platform, or moxibustion technique in order to capture the broad spectrum of microbiota-related evidence associated with moxibustion.

### 2.4 Study selection and data extraction

Two reviewers independently screened records, assessed full texts, and extracted data. Records were managed using NoteExpress. Disagreements were resolved by discussion or consultation with a third reviewer. The study selection process was summarized using a PRISMA flow diagram.

Data were extracted using a predefined form. Extracted information included first author, publication year, study type, disease or model, characteristics of participants or animals, intervention and control details, moxibustion parameters, duration of treatment, microbiome assessment methods, and relevant microbiota, metabolite, and inflammatory outcomes. When numerical data were unavailable from the text or tables, data were extracted from figures using digital measurement tools where appropriate. Attempts were made to contact authors for missing or unclear information when necessary.

When multiple reports appeared to originate from the same study population or experimental cohort, they were treated as a single study. If different reports provided distinct outcomes, the relevant outcome data were extracted separately. If the same outcome was reported repeatedly, the most complete report was used to avoid double counting.

### 2.5 Risk of bias assessment

Risk of bias was assessed independently by two reviewers using a modified SYRCLE risk-of-bias tool for animal studies, with reference to animal research reporting principles from ARRIVE 2.0 where applicable (Hooijmans et al., 2014; Percie et al., 2020). The assessed domains included sequence generation, baseline comparability, allocation concealment, random housing during the experiment, blinding of caregivers and investigators, random outcome assessment, blinding of outcome assessors, incomplete outcome data, selective outcome reporting, and other potential sources of bias. For human studies, animal-specific items, such as random housing during the experiment, were judged as not applicable. Each domain was rated as low risk, high risk, or unclear risk. Disagreements were resolved by discussion or adjudication by a third reviewer.

### 2.6 Data synthesis and statistical analysis

Clinical and preclinical evidence were analyzed separately because of fundamental differences in populations, biological models, intervention contexts, and the availability of comparable outcome data. Quantitative synthesis was undertaken when at least five eligible and sufficiently comparable preclinical animal comparisons were available for an outcome. Outcomes with fewer than five eligible comparisons were retained as limited evidence. Accordingly, the primary quantitative synthesis was limited to preclinical animal studies, and the eligible clinical studies were synthesized narratively.

For microbiota diversity indices and taxonomic abundance outcomes, log-transformed ratios of means (ROMs) and their standard errors were pooled and subsequently back-transformed for presentation. Inflammatory mediators were synthesized using standardized mean differences (SMDs) with 95% confidence intervals. Random-effects models were used because substantial biological and methodological heterogeneity was anticipated. Between-study heterogeneity was assessed using Cochran’s Q and I² statistics. A two-sided P < 0.05 was considered statistically significant for a pooled effect. Final quantitative analyses were conducted in R (version 4.4.2) using the metafor package.

To prevent duplicate weighting of shared control groups, intervention arms within multi-arm studies were combined in the primary analysis when they represented the same moxibustion intervention but differed only in treatment duration, dose, or assessment schedule. A sensitivity analysis was conducted by excluding animal studies judged to have a high overall risk of bias that contributed to a given outcome, to examine the robustness of the pooled estimates. Because “unclear” judgments reflected inadequate reporting rather than confirmed methodological bias, studies were not excluded solely on the basis of multiple unclear domains.

Because most included studies were animal experiments and reporting of sequencing platform, amplified region, moxibustion type, and treatment frequency was often incomplete or unevenly distributed, not all prespecified subgroup analyses were feasible. Subgroup analyses were therefore prioritized for clinically and biologically interpretable dimensions with sufficient data, mainly treatment duration (≤4 weeks vs >4 weeks) and broad disease or model category (intestinal/digestive-related vs non-intestinal-related). Because treatment duration was a potential source of heterogeneity, an additional *post-hoc* exploratory random-effects meta-regression was performed using treatment duration in days as a continuous moderator for outcomes with at least 10 eligible comparisons and sufficient independent study-level information.

### 2.7 Publication bias and certainty of evidence

For prespecified primary microbiota outcomes with at least 10 eligible animal comparisons, funnel plots and Egger’s regression test were used to explore small-study effects. Evidence of funnel-plot asymmetry or a statistically significant Egger test was interpreted as a potential small-study effect. Certainty of evidence for the primary microbiota outcomes: Shannon, Simpson, Firmicutes, Bacteroidetes, *Lactobacillus*, and *Bifidobacterium*, was evaluated using the GRADE framework, considering risk of bias, inconsistency, indirectness, imprecision, and publication bias. For preclinical evidence, particular attention was given to indirectness arising from animal-to-human translation (Hooijmans et al., 2018).

## 3 Results

### 3.1 Study selection and characteristics

The study selection process is shown in **Figure 1**. Consequently, 31 reports representing 30 unique studies were included in the review (An et al., 2024; Bao et al., 2019; Cao et al., 2023; Chen et al., 2024; Fan et al., 2023; Fang et al., 2022; Fu et al., 2024; He et al., 2023; Huang, 2024; Jia et al., 2022; Jing et al., 2021; Li et al., 2021; Lin et al., 2021, 2023, 2024; Ouyang et al., 2022; Pan et al., 2021; Qi et al., 2018, 2022; Song et al., 2024, 2023; Wang et al., 2023, 2022; Wang, 2012; Yang et al., 2024; Yao et al., 2022; Yu et al., 2020; Zhang et al., 2023; Zhao et al., 2024; Zhong et al., 2022; Zhu et al., 2021). These comprised 5 clinical studies and 25 preclinical animal studies. Song et al. (2024) and (2023) were two separate reports originating from the same animal experiment. They were therefore treated as one underlying study for study counting. Because the two reports provided distinct outcomes, relevant non-overlapping data were extracted from each report, while overlapping data were not double counted.

**Figure 1:**
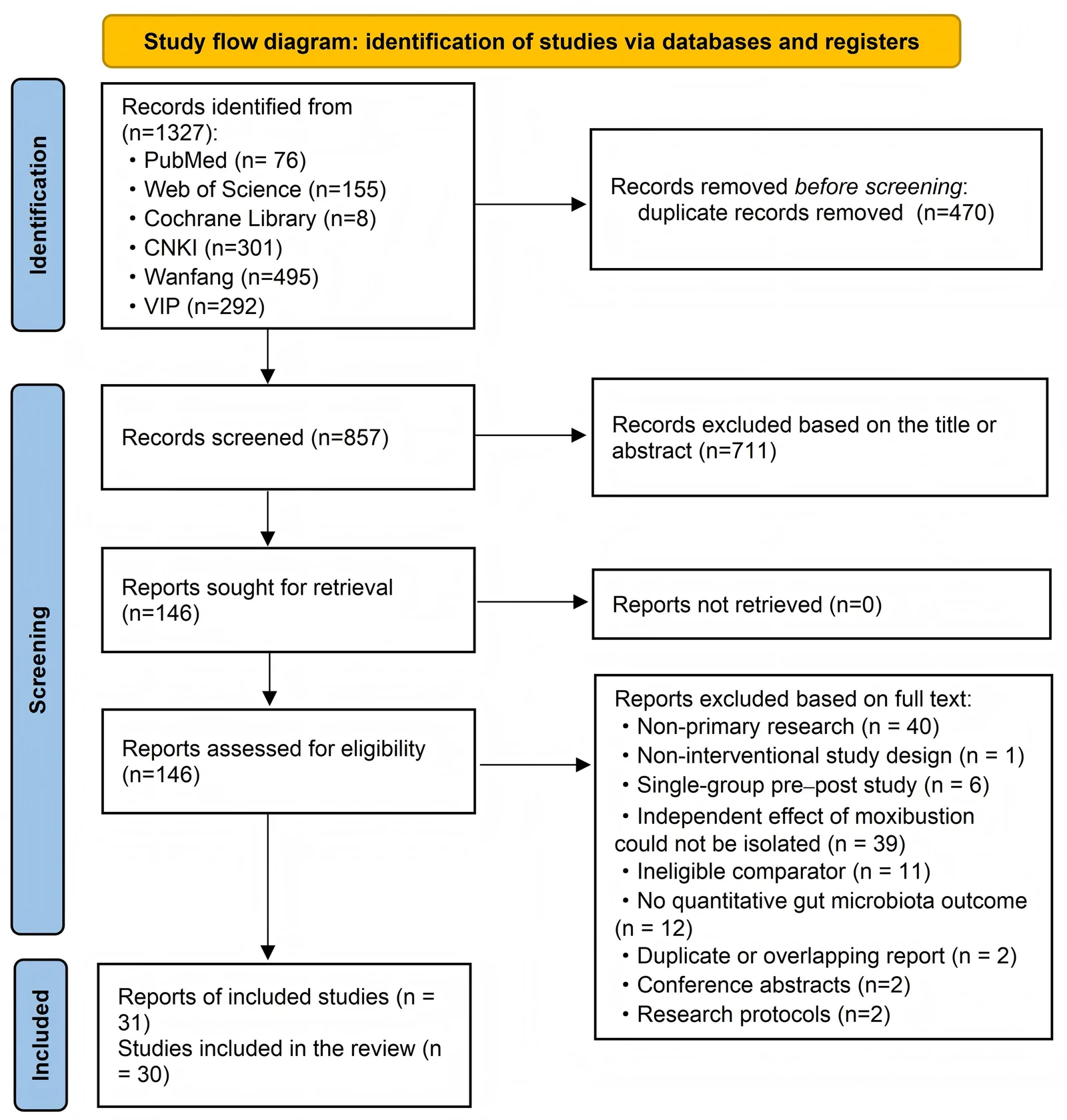
Flow diagram of study selection. CNKI, China National Knowledge Infrastructure.

The included studies were published between 2012 and 2024, and all were conducted in China. The 5 clinical studies involved 555 participants and covered functional constipation in children, gastrointestinal dysfunction during recovery from cerebral infarction, ankylosing spondylitis, phlegm-dampness constitution, and chronic fatigue syndrome. The 25 unique preclinical animal studies included a broad range of disease models, including DSS- or TNBS-induced colitis or Crohn’s disease-like models, colorectal cancer liver metastasis, gastric cancer, intestinal mucositis, irritable bowel syndrome, knee osteoarthritis, polycystic ovary syndrome, aging, depression-like behavior, and spinal cord injury.

The moxibustion interventions mainly included mild moxibustion, ginger-partitioned moxibustion, herbal cake-partitioned moxibustion, and umbilical moxibustion. In all clinical studies, moxibustion was used as an adjunctive therapy combined with the same background treatment as that in the control group. Most animal studies evaluated moxibustion as a standalone intervention and compared it with model, blank, or positive-drug controls. The most frequently used acupoints were Tianshu (ST25) and Zusanli (ST36), followed by Guanyuan (CV4) and Shenque (CV8). Treatment was most commonly administered once daily or every other day, with intervention duration ranging from 7 days to 16 weeks.

Most studies assessed gut microbiota using 16S rRNA or 16S rDNA sequencing. One study used metagenomic sequencing, and 3 studies used targeted or qPCR-based microbial detection. Illumina platforms were the most commonly reported sequencing platforms. Among studies that specified the amplified region, the V3–V4 region was the most frequently used target. Detailed characteristics of the included studies are presented in **Table 1**.

**Table 1:** Characteristics of included studies.

Study	Design	Condition/model	Population/model	Groups/sample size	Moxibustion protocol	Comparator(s)	Microbiota/metabolite assessment
Song et al., 2023, Song et al., 2024*	Preclinical	Colorectal cancer liver metastasis	BALB/c nude mouse model	Model control, prevention, moxibustion, prevention + moxibustion: n = 15/group	Mild moxibustion at BL18 and ST36; 10 min/session, once daily; 5 weeks	Model control; prevention-only and combined schedules	16S rRNA V3-V4 sequencing; Illumina HiSeq 2500; QIIME2
Chen et al., 2024	Clinical	Functional constipation	Children with functional constipation	Moxibustion + herbal medicine: n = 56; herbal medicine: n = 50	Herb-cake-separated umbilical moxibustion at CV8; 2 cones/session, once daily; 7 days	Chinese herbal medicine plus basic treatment	Fecal qPCR for *Lactobacillus*, *Bifidobacterium* and *Escherichia coli*
Zhao et al., 2024	Preclinical	Subacute aging	Rat subacute aging model	Blank, model, ST36 moxibustion: n = 10/group	Non-scarring direct moxibustion at bilateral ST36; 3 cones/point, once daily; 28 days	Blank and model controls	16S rRNA V3-V4 sequencing; Illumina MiSeq; OTU clustering at 97% similarity
An et al., 2024	Clinical	Post-stroke gastrointestinal dysfunction	Patients with gastrointestinal dysfunction during recovery from first cerebral infarction	Moxibustion + domperidone + live combined *Bifidobacterium/Lactobacillus/Enterococcus*: n = 121; same medication/probiotic control: n = 121	Moxibustion at bilateral ST36, BL18 and BL21; 20 min/session, once daily for 5 consecutive days followed by 2 days off; 4 weeks	Domperidone + live combined *Bifidobacterium*, *Lactobacillus* and *Enterococcus*	Fecal culture counts (log CFU/g) for *Bifidobacterium*, *Lactobacillus*, *Enterobacter* and *Enterococcus*
Lin et al., 2024	Preclinical	Polycystic ovary syndrome	DHEA-induced PCOS rat model	Control, PCOS, moxibustion, metformin: n = 9/group	Moxibustion at CV4; 20 min/day; 14 days	Normal control, PCOS model and metformin	16S rDNA V3-V4 sequencing; fecal metabolomics (^1H NMR)
Fu et al., 2024	Preclinical	Osteoarthritis	ACLT-induced osteoarthritis mouse model	Sham, ACLT, ACLT + moxibustion: n = 6/group	Moxibustion at ST36 and BL23; 20 min/session, once daily; 28 days	Sham and ACLT model controls	16S rRNA V3-V4 sequencing; Illumina MiSeq; KEGG pathway analysis
Yang et al., 2024	Preclinical	Inflammatory bowel disease/colitis	DSS-induced IBD mouse model	Control, DSS, DSS + acupuncture, DSS + moxibustion, DSS + mesalazine: n = 10/group	Moxibustion at bilateral ST36 and ST25; 15 min/day; 14 days	Normal, DSS model, acupuncture and mesalazine controls	16S rDNA V3-V4 sequencing and metabolomic pathway analysis
Huang, 2024	Preclinical	Depression	CUMS plus isolation-induced depression rat model	Blank, model, sham vagotomy + moxibustion, vagotomy + moxibustion: n = 8/group	Moxibustion at GV20; 20 min/session, once daily; 3 weeks	Blank and depression model controls; vagotomy-related controls	16S rRNA high-throughput sequencing of colonic contents; platform/region not specified
He et al., 2023	Preclinical	Crohn disease	TNBS-induced CD rat model	Normal n = 10; model, moxibustion, mesalazine: n = 8/group	Moxibustion at bilateral ST25 and ST37; 30 min/session, once daily; 7 days	Normal, model and mesalazine controls	16S rRNA V3-V4 sequencing; Illumina MiniSeq
Cao et al., 2023	Preclinical	Healthy/physiological condition	Healthy SD rats	Blank, acupuncture, moxibustion: n = 11/group	Moxibustion at bilateral ST25 and ST37; 15 min/session, once daily; 7 days	Handling/fixation control and acupuncture control	16S rDNA V3-V4 sequencing; Illumina HiSeq 2500
Fan et al., 2023	Preclinical	Hyperlipidemia	High-cholesterol-diet rabbit model	Normal, model, herb-cake moxibustion: n = 8/group initially; 1 model rabbit died	Herb-cake-separated moxibustion at alternating acupoint sets including ST25, ST40, CV14, BL20, BL15 and BL18; 30 min/session, once daily; 12 weeks	Normal diet and high-cholesterol model controls	16S rDNA V4 sequencing; Illumina platform
Zhang et al., 2023	Preclinical	Natural aging	Young and middle-aged SD rats	Young control n = 10; young moxibustion n = 10; middle-aged control n = 10; middle-aged moxibustion n = 9 final	Moxibustion at GV4 and CV4; 15 min/session, 5 days/week; 4 months	Age-matched non-moxibustion controls	16S rRNA V3-V4 sequencing; Illumina MiSeq
Wang et al., 2023	Clinical	Ankylosing spondylitis	Patients with ankylosing spondylitis and kidney-deficiency/cold pattern	Moxibustion + western medicine n = 45; western medicine n = 45	Ginger-partitioned moxibustion using Lei’s silver cup device at lumbar paraspinal acupoints; once daily; 3 months	Meloxicam plus sulfasalazine	16S rRNA sequencing; Illumina HiSeq; OTU clustering at 97% similarity
Lin et al., 2023	Preclinical	Aging	9-month-old male ICR aging mice	Moxibustion n = 15; control n = 15	Indirect moxibustion at CV4; 20 min/session, every other day; 30 sessions	Fixation without moxibustion	16S rRNA V3-V4 sequencing; Illumina MiSeq
Ouyang et al., 2022	Preclinical	Aging	Aging female Wistar rats versus young controls	Aged + moxibustion, aged control, young control: n = 6/group	Moxibustion at CV4; 20 min/day; 40 days	Aged and young non-moxibustion controls	16S rRNA V3-V4 sequencing; Illumina MiSeq
Wang et al., 2022	Preclinical	Spinal cord injury	Rat SCI model	Sham, SCI model, CV8/GV4 moxibustion, GB34/SP9 moxibustion: n = 8/group	Moxibustion at CV8/GV4 or GB34/SP9; 30 min/session, every other day; 5 sessions	Sham and SCI model controls	16S rRNA V4 sequencing; Illumina NovaSeq 6000
Zhong et al., 2022	Preclinical	Crohn disease	TNBS-induced CD rat model	Normal, model, herb-cake moxibustion, electroacupuncture, sulfasalazine: n = 8/group; microbiome n = 7/group	Herb-cake-separated moxibustion at bilateral ST25 and CV6; 2 cones/point, once daily; 7 days	Normal, model, electroacupuncture and sulfasalazine controls	16S rRNA V3-V4 sequencing; Illumina HiSeq 2500; KEGG functional prediction
Yao et al., 2022	Preclinical	Gut dysbiosis	Antibiotic/LPS-induced gut dysbiosis young mouse model	Blank, model, probiotic, moxibustion, combination: n = 10/group	Moxibustion at CV8, CV12, CV4 and ST25; 15 min/session, once daily; 14 days	Blank, model, probiotic and combination controls	16S rRNA sequencing; Illumina NovaSeq 6000; region not specified
Fang et al., 2022	Preclinical	Hypercholesterolemia	High-cholesterol-diet rabbit model	Blank, model, probiotic, herb-cake moxibustion: n = 9/group	Herb-cake-separated moxibustion at alternating acupoint sets including CV14, ST25, ST40, BL15, BL18 and BL20; once daily; 12 weeks	Blank, high-cholesterol model and probiotics controls	16S rDNA V4 sequencing; Illumina MiniSeq
Jia et al., 2022	Preclinical	Knee osteoarthritis	MIA-induced knee osteoarthritis rat model	Normal n = 6; model n = 5; moxibustion 2, 4 or 6 weeks: n = 5/group	Moxibustion at ST35 and ST36; 30 min/session, every 2 days; 2, 4 or 6 weeks	Normal and MIA model controls	16S rRNA sequencing; Illumina HiSeq 2500; region not specified
Qi et al., 2022	Preclinical	Ulcerative colitis	4% DSS-induced UC rat model	Normal, UC, UC + moxibustion, UC+mesalazine, normal + moxibustion: n = 5/group	Herb-cake-separated moxibustion at bilateral ST25; once daily; 7 days	Normal, UC model, mesalazine and normal+moxibustion controls	Metagenomic sequencing; Illumina HiSeq 2500
Jing et al., 2021	Clinical	Phlegm-dampness constitution	Participants with phlegm-dampness constitution	Umbilical moxibustion + guidance n = 30; guidance n = 30	Herb-cake-separated umbilical moxibustion at CV8; twice weekly; 8 weeks	Diet and exercise guidance	16S rDNA V3-V4 sequencing; Illumina MiSeq
Zhu et al., 2021	Preclinical	Rheumatoid arthritis	Adjuvant arthritis rat model	Normal, model, moxibustion: n = 12/group; molecular analysis n = 4/group	Moxibustion at bilateral ST36 and BL23; 20 min/acupoint, once daily; 21 days	Normal and adjuvant arthritis model controls	16S rRNA sequencing; Illumina MiSeq
Lin et al., 2021	Clinical	Chronic fatigue syndrome	Patients with chronic fatigue syndrome	Moxibustion + lifestyle guidance n = 28; lifestyle guidance n = 29	Ginger-partitioned moxibustion at CV12, CV8 and CV4; 30 min/session, 3 times/week; 4 weeks	Lifestyle guidance	16S rRNA V4 sequencing; Illumina MiSeq 2500
Pan et al., 2021	Preclinical	Gastric cancer	MNNG-induced gastric cancer rat model	Normal, normal + moxibustion, model, model + moxibustion: n = 6/group	Herb-cake-separated moxibustion at ST36, BL21 and CV12; every 3 days; 16 weeks	Normal and model controls with/without moxibustion	16S rRNA V3-V4 sequencing; Illumina HiSeq 2500
Li et al., 2021	Preclinical	Chemotherapy-induced intestinal mucositis	Rat intestinal mucositis model	CON, CHE, MOX, PRO: n = 10/group initially; n = 7/group analyzed	Mild moxibustion at bilateral ST25; 15 min/day; 15 days	Normal, chemotherapy model and probiotic controls	16S rRNA V3-V4 sequencing; MiSeq PE250
Yu et al., 2020	Preclinical	Gastric mucosal injury	Ethanol-induced gastric mucosal injury rat model	Blank, model, meridian acupoint, non-acupoint control: n = 6/group	Moxibustion at Liangmen and ST36 or non-acupoints; 20 min/session, once daily; 7 days	Blank, model and non-acupoint moxibustion controls	16S rRNA V3-V4 paired-end sequencing; Illumina HiSeq 2500
Bao et al., 2019	Preclinical	Post-infectious irritable bowel syndrome	PI-IBS rat model	Normal, model, mild moxibustion, sham moxibustion: n = 10/group	Mild moxibustion at bilateral ST36 and ST25; 10 min/day; 7 days	Normal, model and unlit-moxa sham controls	Fecal qPCR for *Lactobacillus*, *Bifidobacterium*, *Faecalibacterium prausnitzii* and *Escherichia coli*
Qi et al., 2018	Preclinical	Ulcerative colitis	4% DSS-induced UC rat model	Healthy control, healthy+moxibustion 7/14 days, UC, UC + moxibustion 7/14 days, UC+mesalazine: n = 10/group	Herb-cake-separated moxibustion at bilateral ST25; 10 min/session; 7 or 14 days	Healthy and UC controls, with mesalazine comparator	16S rRNA V3-V4 sequencing; Illumina MiSeq PE300
Wang, 2012	Preclinical	Ulcerative colitis	Immunological stimulation-induced UC rat model	Normal, model, herb-partitioned moxibustion, sulfasalazine: n = 10/group	Herb-partitioned moxibustion at ST25 and CV6; once daily; 8 days	Normal, model and sulfasalazine controls	16S rRNA qPCR; target taxa not fully specified

*Song 2023 and Song 2024 were derived from the same underlying animal experiment.

ACLT, anterior cruciate ligament transection; CD, Crohn disease; CFS, chronic fatigue syndrome; CUMS, chronic unpredictable mild stress; DHEA, dehydroepiandrosterone; DSS, dextran sulfate sodium; IBD, inflammatory bowel disease; LPS, lipopolysaccharide; MIA, monoiodoacetate; MNNG, N-methyl-N’-nitro-N-nitrosoguanidine; OTU, operational taxonomic unit; PCOS, polycystic ovary syndrome; PI-IBS, post-infectious irritable bowel syndrome; qPCR, quantitative polymerase chain reaction; SCI, spinal cord injury; TNBS, trinitrobenzene sulfonic acid; UC, ulcerative colitis.

### 3.2 Risk of bias assessment

Risk-of-bias judgments for the 30 unique studies are summarized in **Figures 2A, B**. In general, the methodological quality of the included studies was limited mainly by insufficient reporting of allocation procedures and blinding. By contrast, baseline comparability, completeness of outcome data, and selective outcome reporting were generally better addressed.

**Figure 2:**
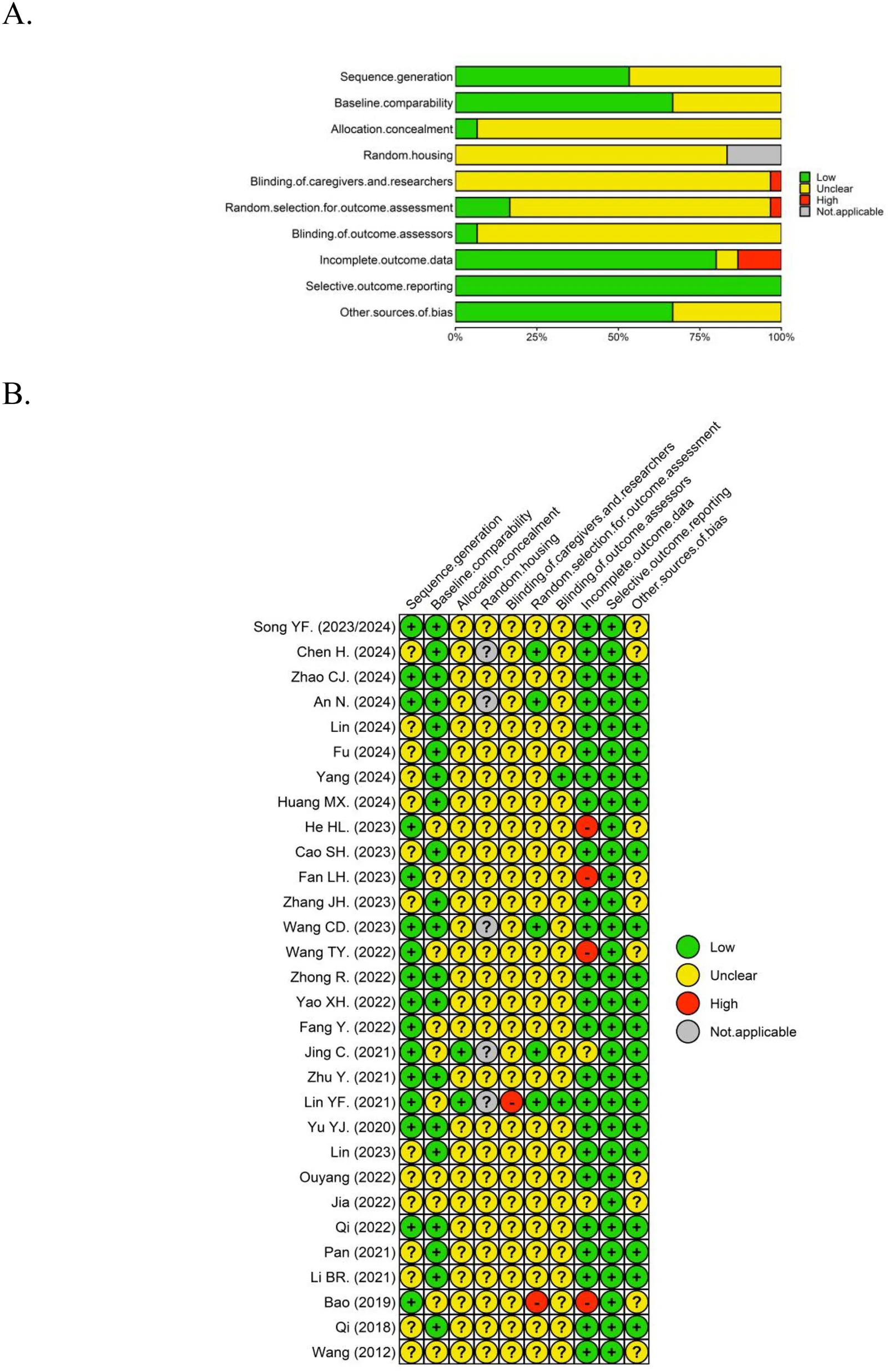
Risk-of-bias assessment of included studies. **(A)** Summary of risk-of-bias judgments across methodological domains. **(B)** Traffic-light plot showing study-level risk-of-bias judgments. Green indicates low risk of bias, yellow indicates unclear risk of bias, red indicates high risk of bias, and grey indicates not applicable.

Allocation concealment was poorly reported. Only 2 studies explicitly described measures to conceal group allocation, whereas the remaining studies provided insufficient information to determine whether assignment could have been predicted before intervention. For the animal-specific domain of random housing during the experiment, none of the animal studies clearly reported random housing or cage allocation procedures.

Blinding was one of the weakest methodological domains. Because of the operational characteristics of moxibustion, blinding of caregivers and investigators is inherently difficult; accordingly, this domain was rarely reported, and no study provided convincing evidence of adequate blinding procedures. Blinding of outcome assessors was also insufficiently described in most studies, with only a small number providing enough information to support a low-risk judgment. In addition, most studies did not clearly report random selection of animals or participants for outcome assessment, making it difficult to exclude potential detection bias.

Overall, five studies were classified as high risk overall, including four preclinical studies (Bao et al., 2019; Fan et al., 2023; He et al., 2023; Wang et al., 2022) and one clinical study (Lin et al., 2021); the remaining studies were classified as unclear.

### 3.3 Quantitative synthesis of preclinical animal evidence

#### 3.3.1 Effects on gut microbiota α-diversity

In the preclinical animal synthesis, moxibustion was associated with significant increases in Chao1 (ROM = 1.079, 95% CI [1.034–1.127], P = 0.0005, I² = 86.8%), ACE (ROM = 1.100, 95% CI [1.044–1.160], P = 0.0004, I² = 73.0%), and Shannon diversity (ROM = 1.047, 95% CI [1.015–1.080], P = 0.0037, I² = 80.7%). Simpson diversity was not significantly altered (ROM = 1.004, 95% CI [0.990–1.019], P = 0.5685, I² = 80.8%) (**Table 2**; **Figure 3**).

**Table 2:** Main animal-only meta-analysis results.

Category	Outcome	k	Effect measure	Pooled effect (95% CI)	P	I² (%)
Alpha diversity	Chao1	18	ROM	1.079 [1.034 to 1.127]	0.0005	86.8
	ACE	10	ROM	1.100 [1.044 to 1.160]	0.0004	73.0
	Shannon	21	ROM	1.047 [1.015 to 1.080]	0.0037	80.7
	Simpson	12	ROM	1.004 [0.990 to 1.019]	0.5685	80.8
Phylum-level	Firmicutes	19	ROM	0.954 [0.843 to 1.080]	0.4608	95.6
	Bacteroidetes	19	ROM	0.956 [0.795 to 1.149]	0.6292	96.3
	Proteobacteria	17	ROM	0.996 [0.634 to 1.563]	0.9847	98.2
	Actinobacteria	10	ROM	0.786 [0.518 to 1.194]	0.2597	97.3
Genus/family-level	*Bacteroides*	17	ROM	0.715 [0.476 to 1.073]	0.1049	98.1
	*Lactobacillus*	21	ROM	0.913 [0.658 to 1.268]	0.5872	98.6
	Lachnospiraceae	8	ROM	0.785 [0.466 to 1.321]	0.3618	96.2
	*Prevotella*	8	ROM	0.761 [0.520 to 1.114]	0.1604	95.3
	*Ruminococcus*	10	ROM	0.643 [0.426 to 0.972]	0.0364	96.9
Inflammatory mediators	TNF-α	9	SMD	-1.648 [-2.659 to -0.637]	0.0014	82.5
	IL-6	7	SMD	-0.665 [-1.448 to 0.118]	0.0961	68.0
	IL-1β	5	SMD	-1.861 [-2.419 to -1.303]	<0.0001	0
	IL-10	7	SMD	0.439 [-0.657 to 1.534]	0.4326	84.3

k, number of comparisons; CI, confidence interval; ROM, ratio of means; SMD, standardized mean difference. Microbiota-related outcomes were synthesized using ROM, whereas inflammatory mediators were synthesized using SMD. All pooled estimates were calculated using random-effects models.

**Figure 3:**
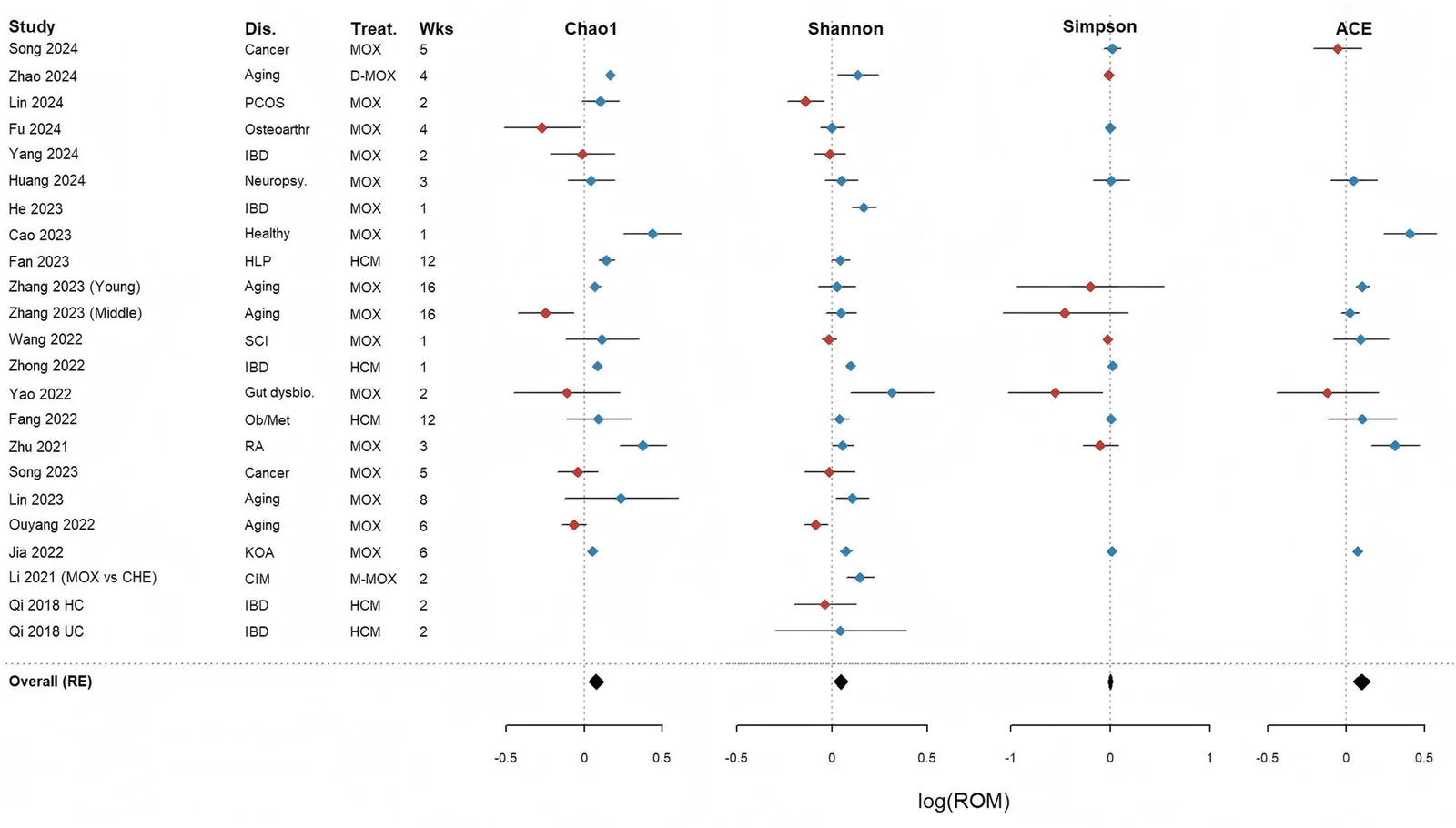
Forest plot of the overall effect of moxibustion on gut microbiota α-diversity. Forest plots show the effects of moxibustion on Chao1, Shannon, Simpson, and ACE indices. Effect sizes are presented as log-transformed ratios of means [log(ROM)]. Each point represents an individual comparison, horizontal lines indicate 95% confidence intervals, and diamonds indicate pooled random-effects estimates. The vertical dashed line represents no effect [log(ROM) = 0]. Values greater than 0 indicate increased α-diversity after moxibustion.

#### 3.3.2 Effects on phylum-level gut microbiota composition

No statistically significant animal-only pooled effect was detected for Firmicutes (ROM = 0.954, 95% CI [0.843–1.080], P = 0.4608, I² = 95.6%), Bacteroidetes (ROM = 0.956, 95% CI [0.795–1.149], P = 0.6292, I² = 96.3%), Proteobacteria (ROM = 0.996, 95% CI [0.634–1.563], P = 0.9847, I² = 98.2%), or Actinobacteria (ROM = 0.786, 95% CI [0.518–1.194], P = 0.2597, I² = 97.3%) (**Table 2**; **Figure 4**).

**Figure 4:**
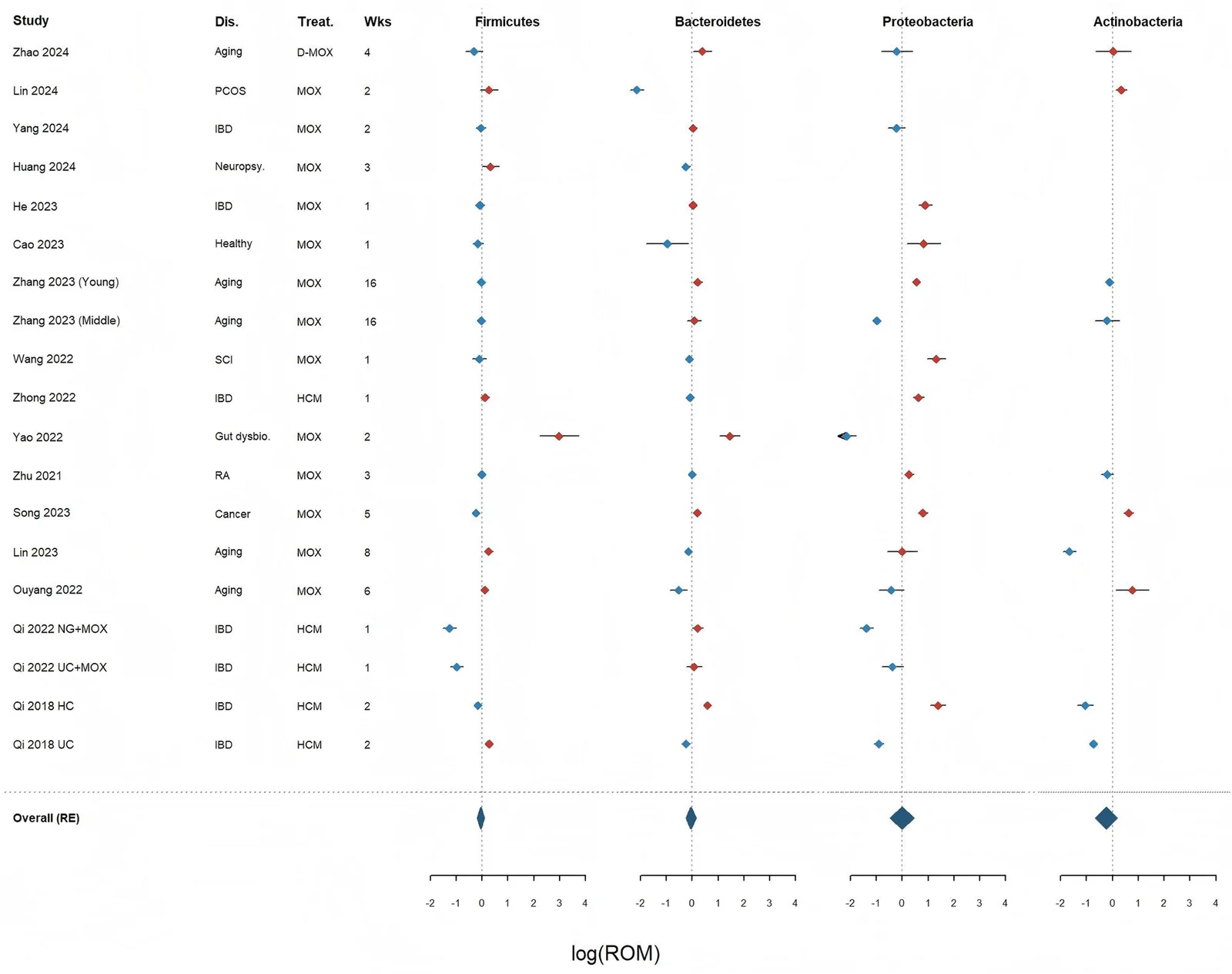
Forest plot of the overall effect of moxibustion on gut microbiota abundance at the phylum level. Forest plots show the effects of moxibustion on the relative abundances of Firmicutes, Bacteroidetes, Proteobacteria, and Actinobacteria. Effect sizes are presented as log-transformed ratios of means [log(ROM)]. Each point represents an individual comparison, horizontal lines indicate 95% confidence intervals, and diamonds indicate pooled random-effects estimates. The vertical dashed line represents no effect [log(ROM) = 0]. Values greater than 0 indicate increased relative abundance after moxibustion, whereas values less than 0 indicate decreased relative abundance.

Verrucomicrobia was supported by only four eligible animal comparisons. Although its exploratory estimate was positive and statistically significant (ROM = 9.567, 95% CI [2.103–43.519]; P = 0.0035), heterogeneity was extreme (I² = 98.7%) (**Supplementary Table S2**).

#### 3.3.3 Effects on genus/family-level gut microbiota composition

Among genus/family-level outcomes retained in the animal-only synthesis, *Ruminococcus* showed a statistically significant reduction (ROM = 0.643, 95% CI [0.426–0.972], P = 0.0364, I² = 96.9%). No statistically significant pooled effects were detected for *Bacteroides* (ROM = 0.715, 95% CI [0.476–1.073], P = 0.1049), *Lactobacillus* (ROM = 0.913, 95% CI [0.658–1.268], P = 0.5872), Lachnospiraceae (ROM = 0.785, 95% CI [0.466–1.321], P = 0.3618), or *Prevotella* (ROM = 0.761, 95% CI [0.520–1.114], P = 0.1604) (**Table 2**; **Figure 5**).

**Figure 5:**
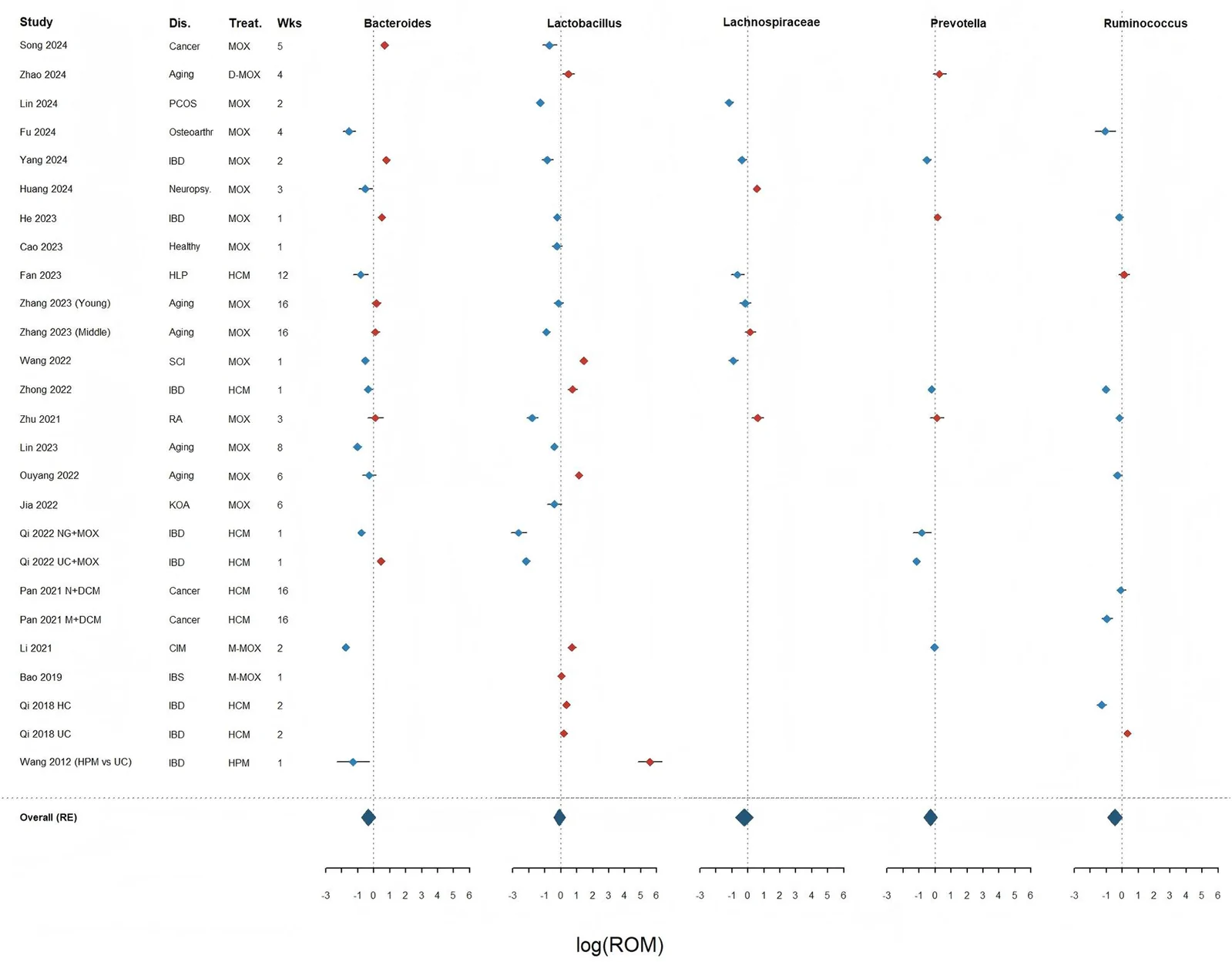
Forest plot of the overall effect of moxibustion on gut microbiota abundance at the genus/family level. Forest plots show the effects of moxibustion on *Bacteroides*, *Lactobacillus*, Lachnospiraceae, *Prevotella*, and *Ruminococcus*. Effect sizes are presented as log-transformed ratios of means [log(ROM)]. Each point represents an individual comparison, horizontal lines indicate 95% confidence intervals, and diamonds indicate pooled random-effects estimates. The vertical dashed line represents no effect [log(ROM) = 0]. Values greater than 0 indicate increased abundance after moxibustion, whereas values less than 0 indicate decreased abundance.

Exploratory estimates were available for *Bifidobacterium* (ROM = 1.183, 95% CI [0.436–3.215]), *Enterobacteriaceae* (ROM = 3.410, 95% CI [2.575–4.516]), *Escherichia* (ROM = 0.408, 95% CI [0.112–1.489]), and *Roseburia* (ROM = 1.053, 95% CI [0.331–3.349]) (**Supplementary Table S2**).

#### 3.3.4 Effects on inflammatory mediators

Moxibustion significantly reduced TNF-α (SMD = −1.648, 95% CI [−2.659 to −0.637], P = 0.0014, I² = 82.5%) and IL-1β (SMD = −1.861, 95% CI [−2.419 to −1.303], P < 0.0001, I² = 0%). For IL-6, the pooled estimate was directionally negative but did not reach statistical significance (SMD = −0.665, 95% CI [−1.448 to 0.118], P = 0.0961, I² = 68.0%). No statistically significant pooled effect was detected for IL-10 (SMD = 0.439, 95% CI [−0.657 to 1.534], P = 0.4326, I² = 84.3%) (**Table 2**; **Figure 6**).

**Figure 6:**
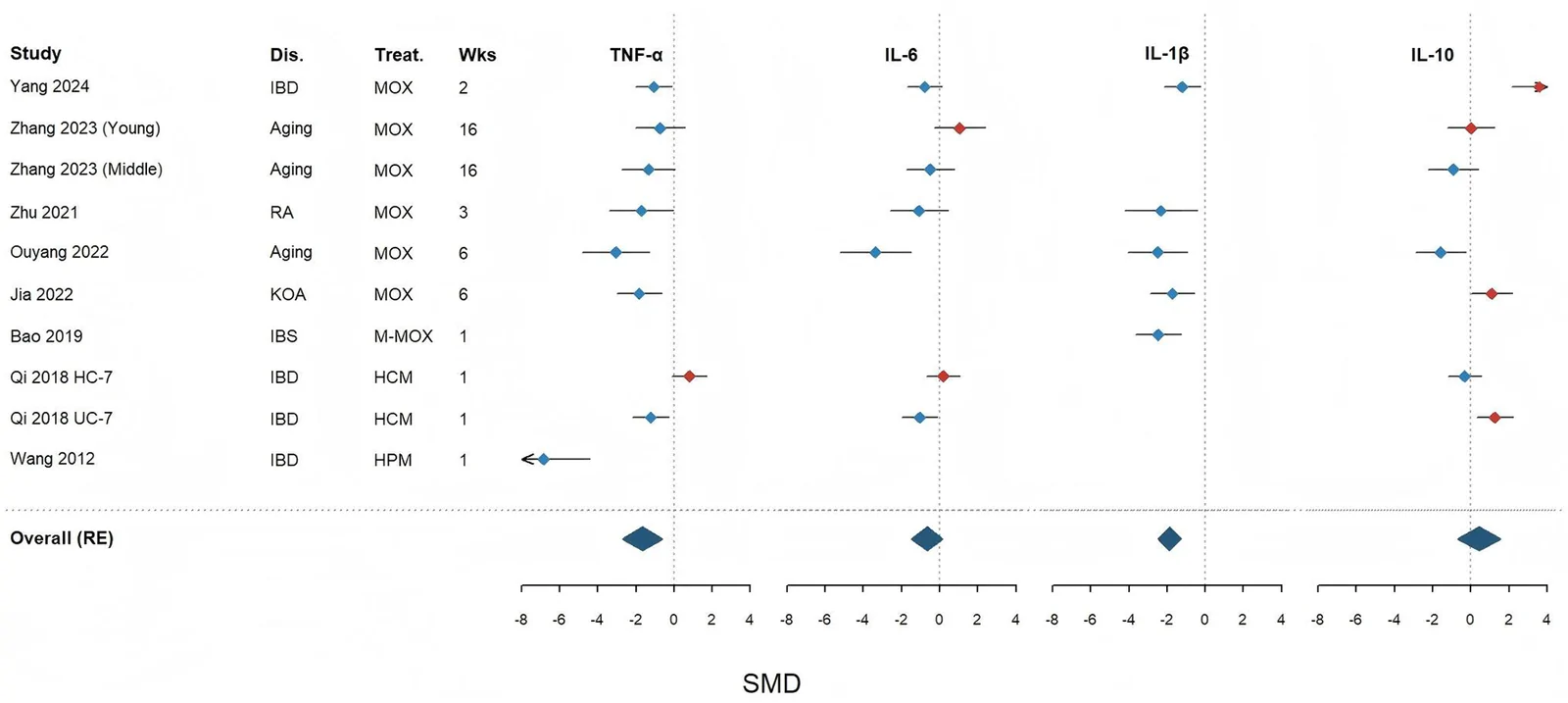
Forest plot of the overall effect of moxibustion on inflammatory mediators. Forest plots show the effects of moxibustion on TNF-α, IL-6, IL-1β, and IL-10. Effect sizes are presented as standardized mean differences (SMDs). Each point represents an individual comparison, horizontal lines indicate 95% confidence intervals, and diamonds indicate pooled random-effects estimates. The vertical dashed line represents no effect (SMD = 0). Negative values indicate lower inflammatory mediator levels after moxibustion.

Only three eligible animal comparisons were available for IL-17. The exploratory estimate was SMD = −0.478 (95% CI [−1.115 to 0.158]; P = 0.1407; I² = 0.0%) (**Supplementary Table S2**).

#### 3.3.5 Sensitivity analyses

Risk-of-bias sensitivity analyses are presented in **Supplementary Table S3**. Exclusion of animal studies (Bao et al., 2019; Fan et al., 2023; He et al., 2023; Wang et al., 2022) with an explicit high-risk judgment did not change the statistical significance status of any principal outcome. The increases in Chao1 (ROM = 1.071, 95% CI [1.022–1.122]; P = 0.0040), ACE (ROM = 1.101, 95% CI [1.042–1.164]; P = 0.0007), and Shannon diversity (ROM = 1.044, 95% CI [1.009–1.080]; P = 0.0128) remained statistically significant. Simpson diversity remained non-significant after exclusion of Wang 2022 (ROM = 1.011, 95% CI [0.999–1.023]; P = 0.0639).

The significant overall reduction in *Ruminococcus* was also retained after exclusion of high-risk studies (ROM = 0.578, 95% CI [0.348–0.960]; P = 0.0343). The TNF-α estimate was unchanged because no contributing study met the high-risk exclusion criterion for that outcome, whereas the reduction in IL-1β remained significant after exclusion of Bao 2019 (SMD = −1.691, 95% CI [−2.323 to −1.059]; P < 0.0001). No principal outcome changed its statistical significance status.

#### 3.3.6 Subgroup analyses and treatment-duration meta-regression

Exploratory animal-only subgroup analyses according to treatment duration (≤ 4 weeks vs > 4 weeks) and model (intestinal/digestive-related vs non-intestinal-related) background are summarized in **Table 3**.

**Table 3:** Animal-only subgroup analyses of selected outcomes.

Domain	Outcome	Duration subgroup results	model subgroup results	Interpretation
α-diversity	Chao1	≤4 weeks: k = 11, ROM = 1.120 [1.056 to 1.187], P = 0.0002, I² = 89.3%; >4 weeks: k = 8, ROM = 1.036 [0.984 to 1.091], P = 0.1798, I² = 80.1%	Intestinal/digestive-related: k = 4, ROM = 1.027 [0.940 to 1.123], P = 0.5548, I² = 45.4%; Non-intestinal-related: k = 14, ROM = 1.094 [1.036 to 1.155], P = 0.0012, I² = 89.4%	Increased significantly in ≤4-week and non-intestinal subgroups.
	ACE	≤4 weeks: k = 6, ROM = 1.159 [1.021 to 1.316], P = 0.0226, I² = 81.2%; >4 weeks: k = 5, ROM = 1.071 [1.033 to 1.111], P = 0.0002, I² = 51.7%	Intestinal/digestive-related: k = 2, ROM = 0.934 [0.813 to 1.074], P = 0.3379, I² = 0.0%; Non-intestinal-related: k = 8, ROM = 1.120 [1.060 to 1.182], P < 0.0001, I² = 76.0%	Increased significantly in both duration strata; by disease background, the significant increase was confined to non-intestinal models.
	Shannon	≤4 weeks: k = 14, ROM = 1.060 [1.019 to 1.103], P = 0.0040, I² = 82.9%; >4 weeks: k = 8, ROM = 1.031 [0.991 to 1.072], P = 0.1277, I² = 62.9%	Intestinal/digestive-related: k = 8, ROM = 1.095 [1.036 to 1.157], P = 0.0012, I² = 68.5%; Non-intestinal-related: k = 13, ROM = 1.027 [0.993 to 1.062], P = 0.1184, I² = 74.8%	Increase remained significant in ≤4-week and intestinal/digestive-related subgroups; >4-week and non-intestinal subgroups were not significant.
	Simpson	≤4 weeks: k = 8, ROM = 1.003 [0.984 to 1.021], P = 0.7894, I² = 85.8%; >4 weeks: k = 5, ROM = 1.010 [1.002 to 1.018], P = 0.0108, I² = 0.0%	Intestinal/digestive-related: k = 3, ROM = 1.005 [0.920 to 1.098], P = 0.9087, I² = 64.7%; Non-intestinal-related: k = 9, ROM = 0.999 [0.985 to 1.014], P = 0.9418, I² = 65.4%	Overall animal-only effect null; an isolated small signal appeared in the >4-week subgroup.
Phylum	Bacteroidetes	≤4 weeks: k = 14, ROM = 0.946 [0.731 to 1.223], P = 0.6699, I² = 97.0%; >4 weeks: k = 5, ROM = 0.993 [0.802 to 1.231], P = 0.9510, I² = 89.5%	Intestinal/digestive-related: k = 9, ROM = 1.236 [1.028 to 1.487], P = 0.0244, I² = 93.9%; Non-intestinal-related: k = 10, ROM = 0.726 [0.520 to 1.013], P = 0.0593, I² = 96.7%	No duration subgroup effect; intestinal/digestive-related models increased significantly, while non-intestinal models showed a non-significant downward direction.
Genus	*Bacteroides*	≤4 weeks: k = 11, ROM = 0.656 [0.386 to 1.116], P = 0.1202, I² = 98.2%; >4 weeks: k = 6, ROM = 0.833 [0.453 to 1.533], P = 0.5577, I² = 97.0%	Intestinal/digestive-related: k = 8, ROM = 0.833 [0.425 to 1.635], P = 0.5960, I² = 98.9%; Non-intestinal-related: k = 9, ROM = 0.621 [0.440 to 0.876], P = 0.0066, I² = 91.4%	Significant decrease was observed only in non-intestinal models, with very high heterogeneity.
	*Prevotella*	≤4 weeks: k = 8, ROM = 0.761 [0.520 to 1.114], P = 0.1604, I² = 95.3%; >4 weeks: no eligible comparison	Intestinal/digestive-related: k = 6, ROM = 0.660 [0.424 to 1.028], P = 0.0662, I² = 96.4%; Non-intestinal-related: k = 2, ROM = 1.212 [0.897 to 1.638], P = 0.2113, I² = 0.0%	No statistically robust pooled subgroup effect was identified.
	*Ruminococcus*	≤4 weeks: k = 6, ROM = 0.582 [0.315 to 1.075], P = 0.0837, I² = 98.0%; >4 weeks: k = 4, ROM = 0.744 [0.488 to 1.133], P = 0.1684, I² = 86.6%	Intestinal/digestive-related: k = 6, ROM = 0.594 [0.318 to 1.111], P = 0.1030, I² = 98.2%; Non-intestinal-related: k = 4, ROM = 0.776 [0.568 to 1.059], P = 0.1105, I² = 71.0%	Neither duration nor disease/model subgroup reached statistical significance.
Inflammatory marker	TNF-α	≤4 weeks: k = 6, SMD= -1.630 [-2.998 to -0.262], P = 0.0195, I² = 87.2%; >4 weeks: k = 4, SMD= -1.862 [-2.986 to -0.737], P = 0.0012, I² = 52.0%	Intestinal/digestive-related: k = 4, SMD= -1.762 [-3.737 to 0.212], P = 0.0802, I² = 91.9%; Non-intestinal-related: k = 5, SMD= -1.624 [-2.312 to -0.936], P < 0.0001, I² = 14.5%	Reduction was observed in both duration strata and was more robust in non-intestinal models.
	IL-1β	≤4 weeks: k = 4, SMD= -1.701 [-2.328 to -1.074], P < 0.0001, I² = 6.3%; >4 weeks: k = 2, SMD= -2.184 [-3.287 to -1.082], P = 0.0001, I² = 0.0%	Intestinal/digestive-related: k = 2, SMD= -1.787 [-3.010 to -0.564], P = 0.0042, I² = 61.1%; Non-intestinal-related: k = 3, SMD= -2.054 [-2.892 to -1.217], P < 0.0001, I² = 0.0%	Reduction remained significant across both duration and disease/model strata, although some strata contained few comparisons.

ROM, ratio of means; SMD, standardized mean difference; CI, confidence interval.

For α-diversity indices, Chao1 increased significantly in the ≤4 weeks subgroup (ROM = 1.120, 95% CI [1.056–1.187]; P = 0.0002), whereas the >4 weeks subgroup showed a non-significant upward trend (ROM = 1.036, 95% CI [0.984–1.091]; P = 0.1798). By model background, Chao1 increased in non-intestinal models (ROM = 1.094, 95% CI [1.036–1.155]; P = 0.0012), but not in intestinal/digestive-related models. ACE increased in both duration strata ≤4 weeks (ROM = 1.159, 95% CI [1.021–1.316]; P = 0.0226) and >4 weeks (ROM = 1.071, 95% CI [1.033–1.111]; P = 0.0002), whereas the disease/model analysis showed an increase in non-intestinal models (ROM = 1.120, 95% CI [1.060–1.182]; P < 0.0001) but not in intestinal/digestive-related models. Shannon increased in the ≤4 week stratum (ROM = 1.060, 95% CI [1.019–1.103]; P = 0.0040) and in intestinal/digestive-related models (ROM = 1.095, 95% CI [1.036–1.157]; P = 0.0012), whereas the corresponding >4 week and non-intestinal estimates were not significant. Simpson remained null overall; an isolated small increase was observed in the >4 week subgroup (ROM = 1.010, 95% CI [1.002–1.018]; P = 0.0108), but neither model subgroup was significant.

At the phylum/genus level of gut microbiota, Bacteroidetes showed no significant pattern by duration. In the model analysis, however, Bacteroidetes increased in intestinal/digestive-related models (ROM = 1.236, 95% CI [1.028–1.487]; P = 0.0244), whereas the estimate in non-intestinal models was directionally lower but did not reach statistical significance (ROM = 0.726, 95% CI [0.520–1.013]; P = 0.0593). *Bacteroides* showed no significant duration-specific effect, but a reduction was observed in non-intestinal models (ROM = 0.621, 95% CI [0.440–0.876]; P = 0.0066). No statistically significant subgroup estimate was identified for *Prevotella*. Although *Ruminococcus* was significantly reduced in the overall animal-only analysis, neither duration stratum nor disease/model stratum independently reached statistical significance.

For inflammatory mediators, TNF-α decreased significantly in both the ≤4 week (SMD = −1.630, 95% CI [−2.998 to −0.262]; P = 0.0195) and >4 week strata (SMD = −1.862, 95% CI [−2.986 to −0.737]; P = 0.0012). By model background, the reduction was significant in non-intestinal models (SMD = −1.624, 95% CI [−2.312 to −0.936]; P < 0.0001), whereas the intestinal/digestive-related estimate did not reach significance. IL-1β decreased significantly in both duration strata and in both disease/model strata, although several of these strata contained only two to four comparisons.

Continuous treatment-duration meta-regression was feasible for eight outcomes after applying the data-sufficiency criterion: Chao1, Shannon, Simpson, Firmicutes, Bacteroidetes, Proteobacteria, *Bacteroides*, and *Lactobacillus*. No statistically significant duration moderator was identified for any of these outcomes (all P > 0.05; **Supplementary Table S4**), and the R² analogue was 0% in each fitted model. Thus, although several categorical duration strata showed statistically significant within-stratum effects, the continuous meta-regression provided no evidence that treatment duration alone explained between-study variation in effect estimates. Cumulative moxibustion exposure and other study-level moderators could not be modeled reliably because the necessary outcome-specific intervention and study-characteristic data were incomplete or unevenly distributed.

### 3.4 Clinical evidence: narrative synthesis

No eligible clinical study in the evidence set provided a usable Shannon or Simpson outcome. For Firmicutes, three clinical studies comprising 207 analyzed participants provided study-specific ROMs of 0.935, 1.068, and 1.053, with estimates close to the null and no consistent directional effect. The same three studies reported Bacteroidetes, with study-specific ROMs of 0.981, 1.053, and 0.909, again showing no consistent direction.

*Lactobacillus* was reported quantitatively in three clinical studies comprising 438 analyzed participants. Two studies showed higher levels with moxibustion-containing treatment (ROMs 1.084 and 1.209), whereas one showed a decrease (ROM 0.758), indicating substantial directional inconsistency. *Bifidobacterium* was reported across all five clinical studies comprising 555 analyzed participants, although only four provided extractable quantitative estimates. The quantitative estimates were 1.090, 1.208, 1.120, and 0.368, while the remaining study reported a non-significant increasing tendency.

### 3.5 Metabolite- and pathway-related findings

Three studies reported metabolite- or pathway-related outcomes (Lin et al., 2024; Yang et al., 2024; Zhong et al., 2022). Owing to substantial heterogeneity in analytical platforms, disease models, and reporting metrics, quantitative pooling was not performed; the evidence was synthesized narratively.

In rat models of polycystic ovary syndrome, Lin et al. (2024) found that moxibustion increased several fecal metabolites, including the short-chain fatty acids acetate, propionate, and butyrate, as well as multiple amino acids. Pathway enrichment suggested involvement of amino acid metabolism, carbohydrate metabolism, and translation-related processes, with the overall fecal metabolic profile shifting toward that of the control group. Yang et al. (2024) reported that acupuncture and moxibustion shared 16 metabolic pathways in an inflammatory bowel disease model, whereas moxibustion showed broader intervention-specific pathway regulation, including taurine and hypotaurine metabolism, valine, leucine, and isoleucine biosynthesis, and HIF-1 signaling, suggesting modality-specific metabolic effects. In Zhong et al. (2022), 16S rDNA-based KEGG functional prediction indicated that several microbial functional pathways were reduced in Crohn’s disease model rats; after moxibustion, pathways related to membrane transport, carbohydrate metabolism, amino acid metabolism, replication and repair, and translation showed a trend toward restoration.

### 3.6 Small-study effects and publication bias

Small-study effects were assessed for the prespecified primary microbiota outcomes when at least 10 eligible animal comparisons were available (**Figure 7**; **Supplementary Table S5**). Egger’s regression test did not indicate significant funnel-plot asymmetry for Shannon diversity (P = 0.2698), Firmicutes (P = 0.7849), Bacteroidetes (P = 0.3576), or *Lactobacillus* (P = 0.5793). In contrast, significant funnel-plot asymmetry was detected for Simpson diversity (P = 0.0125), suggesting possible small-study effects for this outcome.

**Figure 7:**
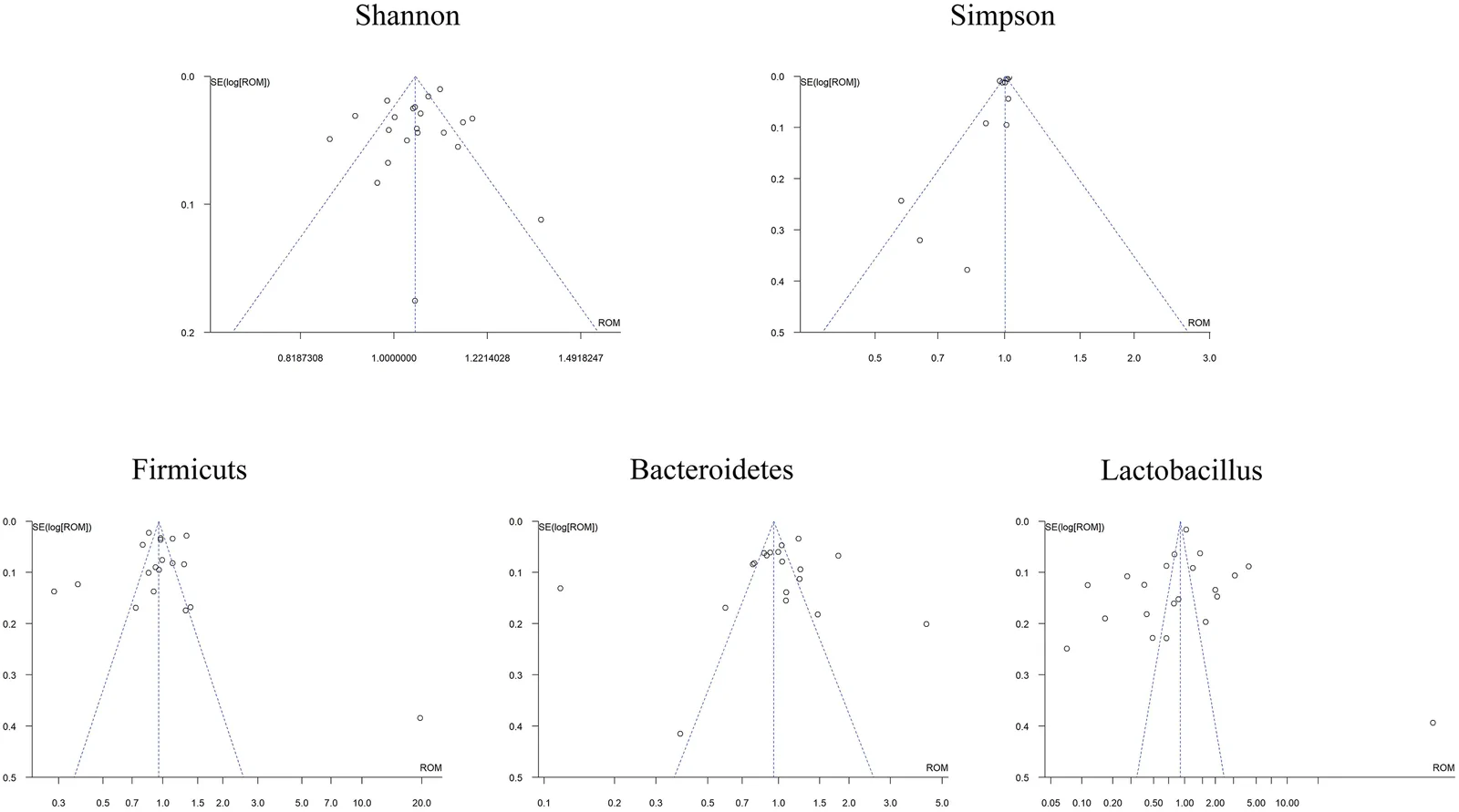
Funnel plots in Shannon diversity, Simpson diversity, Firmicutes, Bacteroidetes, and *Lactobacillus*. AS, ankylosing spondylitis; CI, confidence interval; CFS, chronic fatigue syndrome; CIM, chemotherapy-induced intestinal mucositis; D-MOX, direct moxibustion; Dis., disease/model category; GPM, ginger-partitioned moxibustion; HCM, herb-cake-separated moxibustion; HLP, hyperlipidemia; HPM, herb-partitioned moxibustion; IBD, inflammatory bowel disease; IBS, irritable bowel syndrome; IL, interleukin; M-MOX, mild moxibustion; MOX, moxibustion; Ob/Met, obesity/metabolic disorder; PCOS, polycystic ovary syndrome; PDC, phlegm-dampness constitution; ROM, ratio of means; SCI, spinal cord injury; SMD, standardized mean difference; TNF-α, tumor necrosis factor-α; Treat., treatment; UMB, umbilical moxibustion; Wks, weeks.

### 3.7 Certainty of evidence

The certainty of evidence for the six prespecified primary microbiota outcomes was evaluated separately for the preclinical and clinical evidence bodies using the GRADE framework (**Supplementary Table S6**). For the preclinical evidence, the certainty was rated as very low for Shannon diversity, Simpson diversity, Firmicutes, Bacteroidetes, *Lactobacillus*, and *Bifidobacterium*. The principal reasons for downgrading were risk-of-bias concerns, substantial or very substantial between-study inconsistency, indirectness arising from heterogeneous animal species and disease models, and, for several outcomes, imprecision. Simpson diversity was additionally downgraded for suspected small-study effects.

The clinical evidence was also limited in certainty. No eligible clinical study in the evidence set provided a usable Shannon or Simpson outcome; these outcomes were therefore not graded. For Firmicutes and Bacteroidetes, three randomized clinical studies comprising 207 analyzed participants contributed outcome-specific evidence, but study-level effects were inconsistent and insufficiently precise, resulting in very-low-certainty ratings. *Lactobacillus* was reported quantitatively in three clinical studies comprising 438 participants, resulting in very-low-certainty evidence because of marked inconsistency and risk-of-bias concerns. *Bifidobacterium* was reported across five clinical studies comprising 555 participants, although only four provided extractable quantitative effects; findings were directionally inconsistent across studies and the certainty was likewise rated as very low.

## 4 Discussion

To our knowledge, this is the first systematic review and meta-analysis to evaluate the independent effects of moxibustion on gut microbiota-related outcomes and inflammatory mediators across clinical and preclinical studies. The primary quantitative synthesis was restricted to animal studies because the clinical evidence was too sparse and heterogeneous for stable outcome-level pooling. In the animal evidence, the most consistent microbiota-related findings were increases in richness-related or richness-sensitive α-diversity indices, including Chao1, ACE, and Shannon, whereas Simpson diversity was not significantly altered. No stable pooled effects were observed for the major phyla. At the genus/family level, *Ruminococcus* was significantly decreased, whereas *Bacteroides*, *Lactobacillus*, Lachnospiraceae, and *Prevotella* did not show statistically significant pooled effects. In parallel, moxibustion reduced key pro-inflammatory mediators, including TNF-α and IL-1β, but did not significantly alter IL-6 or IL-10. Several additional outcomes, including Verrucomicrobia, *Bifidobacterium*, *Enterobacteriaceae*, *Escherichia*, *Roseburia*, and IL-17, were supported by too few eligible animal comparisons to meet the prespecified threshold for principal quantitative synthesis and were therefore retained as limited evidence.

These findings are biologically meaningful because they suggest that the gut-related effects of moxibustion may be better understood as partial remodeling of host–microbiota inflammatory networks rather than broad “normalization” of microbiota composition. Notably, these findings do not support a universal taxonomic signature of moxibustion. Therefore, the present findings should be interpreted as evidence of a potential microbiota–inflammation regulatory pattern. Furthermore, the clinical evidence remains insufficient to determine whether the microbiota-related patterns observed in animal models are reproducible in humans, and the overall certainty of evidence for the prespecified primary microbiota outcomes was very low.

The selective pattern observed here differs from that reported by Bae et al., whose synthesis combined acupuncture and/or moxibustion studies and found increased Simpson diversity, Firmicutes, *Lactobacillus*, and *Ruminococcus* together with decreased Bacteroidetes (Bae et al., 2024). In contrast, by analyzing moxibustion as an independent intervention, the present review found a more focused and heterogeneous signal. Specifically, the moxibustion-only animal analysis identified increases in Chao1, ACE, and Shannon without stable pooled changes in Firmicutes, Bacteroidetes, or *Lactobacillus*. This difference suggests that the microbiota profile inferred from a combined acupuncture–moxibustion evidence base may not be directly attributable to moxibustion itself. In addition, the differences in study populations, disease models, and evidence composition may also have contributed to the contrasting pooled results. This distinction is clinically and mechanistically relevant. Yang et al. (2024) reported that acupuncture and moxibustion regulate gut microbiota and metabolic pathways in overlapping but non-identical ways in DSS-induced inflammatory bowel disease, with moxibustion affecting a broader range of metabolism-related pathways. Thus, separating moxibustion from acupuncture is necessary for identifying intervention-specific microbiota–immune signatures. From a mechanistic perspective, thermal stimulation, sensory input, local tissue responses, and neuroimmune signaling may differ between these modalities, and these differences may translate into distinct downstream effects on microbial ecology and inflammatory pathways. Nevertheless, direct head-to-head mechanistic studies are still required before these modality-specific pathways can be regarded as established.

Chao1 and ACE primarily reflect microbial richness, whereas the Shannon index incorporates both richness and abundance distribution, and the Simpson index is more strongly influenced by dominance and evenness (Cassol et al., 2025). Therefore, the increases in Chao1, ACE, and Shannon observed in this study, together with the unchanged Simpson diversity, suggest that moxibustion may preferentially increase detectable microbial taxa and partially restore community complexity, rather than induce a complete restructuring of dominant community members. Because α-diversity is an ecological descriptor rather than a direct measure of microbiome health, however, increased diversity should not itself be interpreted as uniformly beneficial. Its biological meaning depends on the baseline disease-associated ecosystem and should be considered together with taxonomic, metabolic, and inflammatory changes.

At the phylum level, the lack of stable pooled effects on Firmicutes, Bacteroidetes, Proteobacteria, and Actinobacteria should not be interpreted as absence of microbiota modulation. Broad phylum-level markers, especially the Firmicutes/Bacteroidetes ratio, are increasingly recognized as insufficiently specific because they are influenced by host genetics, diet, disease context, sequencing workflow, and bioinformatic pipelines (Magne et al., 2020; Van Hul et al., 2024).

An exploratory increase in Verrucomicrobia was observed, but this finding was based on only four eligible comparisons and showed extreme heterogeneity. The best-characterized member of this phylum, *Akkermansia muciniphila*, is a mucin-associated commensal involved in mucus-layer ecology and barrier and immune regulation (Mo et al., 2024; Shaheen et al., 2025). However, a phylum-level Verrucomicrobia signal cannot be equated with a specific increase in *A. muciniphila*. Thus, *A. muciniphila* should be regarded as a biologically plausible candidate for future investigation rather than as a microbial mediator demonstrated by the present meta-analysis.

At the genus/family level, *Ruminococcus* was the only taxon retained in the principal animal synthesis that showed a statistically significant pooled decrease. However, the very high heterogeneity (I² = 96.9%) and the functional diversity within this taxonomic group require cautious interpretation. *Ruminococcus gnavus* has also received increasing attention because of its association with intestinal and extraintestinal inflammatory diseases (Hong et al., 2024). A recent review highlights that *R. gnavus* is functionally heterogeneous and may be linked to mucus utilization, carbohydrate metabolism, inflammatory metabolites, and strain-specific disease associations (Crost et al., 2023). Accordingly, the pooled decrease in genus-level *Ruminococcus* cannot be assumed to represent a reduction in *R. gnavus* specifically, nor can it be interpreted as uniformly beneficial across disease models. *Escherichia* was supported by only four eligible animal comparisons and its exploratory estimate was not statistically significant. Nevertheless, one included study (Bao et al., 2019) used targeted qPCR and reported *Escherichia coli* together with *Faecalibacterium prausnitzii*. Species-level measurements were also reported in the clinical evidence (Chen et al., 2024), including targeted quantification of *E. coli*, but such data were too sparse and methodologically heterogeneous for cross-study quantitative synthesis. Importantly, the biological behavior of pathogenic or adherent-invasive *E. coli* cannot be inferred from aggregate *Escherichia* abundance. Expansion of Enterobacteriaceae, including adherent-invasive *Escherichia coli* in specific contexts, has been linked to dysbiosis, epithelial barrier dysfunction, and IBD pathogenesis (Gilliland et al., 2024; Mirsepasi-Lauridsen et al., 2019). Therefore, species- and strain-resolved sequencing or targeted quantification is required before individual bacterial lineages can be placed mechanistically between moxibustion and inflammatory regulation. *Prevotella* similarly showed no statistically significant animal-only pooled effect. *Prevotella* is also context dependent: some species or strains may support mucosal function, whereas others may promote inflammation depending on host and ecological background (Wang et al., 2026). Accordingly, the present review does not support describing *Prevotella* reduction as a confirmed effect of moxibustion. More broadly, the contrast between genus-level heterogeneity and the limited species-level information available in the included studies indicates that taxonomic resolution is currently a major constraint on mechanistic interpretation. The absence of stable increases in *Lactobacillus* and *Bifidobacterium* is also informative. Although both genera are generally regarded as beneficial bacteria, and several included studies reported their enrichment in specific models such as colitis, osteoarthritis, and polycystic ovary syndrome (Fu et al., 2024; Li et al., 2021; Lin et al., 2024; Qi et al., 2018, 2022), *Lactobacillus* showed no significant pooled effect in the animal synthesis, whereas *Bifidobacterium* was supported by only four eligible comparisons and therefore did not meet the threshold for principal pooling. The available clinical studies also showed inconsistent directions for both taxa. These findings therefore suggest that the microbiota-related benefits of moxibustion may depend on the restoration of ecological and functional relationships.

The pooled reductions in TNF-α and IL-1β provide an immunological counterpart to the microbiota findings. TNF-α and IL-1β are key pro-inflammatory mediators involved in intestinal inflammatory amplification and barrier dysfunction (Garlanda et al., 2025; Neurath, 2024). Barrier disruption allows microbial products and luminal antigens to amplify local and systemic inflammation (Neurath et al., 2025). IL-1β, as a major inflammasome-related cytokine, contributes to intestinal inflammation, immune-cell recruitment, Th17 differentiation, and barrier disruption (Garlanda et al., 2025). Importantly, the reductions in TNF-α and IL-1β remained statistically significant in the risk-of-bias sensitivity analyses, supporting the robustness of these two inflammatory signals to exclusion of explicitly high-risk studies where applicable. In contrast, IL-17 was supported by only three eligible animal comparisons and its pooled estimate was not statistically significant; it should therefore be regarded as limited evidence rather than a confirmed anti-inflammatory effect of moxibustion. IL-6 is a pleiotropic cytokine whose expression varies by disease stage, tissue source, and sampling time (Swaroop et al., 2024). IL-10 is a compensatory anti-inflammatory cytokine, and its expression may increase or decrease depending on disease activity and host immune status (Branchett et al., 2024). The IL-6 estimate was directionally negative but imprecise, whereas IL-10 showed substantial between-study heterogeneity; these two non-significant findings therefore should not be interpreted identically. The included studies assessed cytokines at different end points across acute and chronic models, which may correspond to different phases of inflammatory initiation, peak activation, compensatory regulation, or resolution. IL-6 may predominate during active inflammatory amplification, whereas IL-10 may vary as a counter-regulatory response according to preceding inflammatory burden and sampling time. Because sampling time was not standardized across studies, however, these phase-related explanations remain biologically plausible interpretations rather than mechanisms demonstrated by the present meta-analysis. Consequently, a single terminal measurement may miss transient or phase-specific cytokine responses. Longitudinal sampling will be required to determine whether moxibustion changes the temporal trajectories of these mediators rather than their concentration at a single endpoint. Overall, the inflammatory results suggest that moxibustion may preferentially suppress excessive pro-inflammatory activation rather than uniformly shift all cytokines toward an anti-inflammatory profile.

Subgroup analyses further explored whether moxibustion-related microbiota responses varied according to treatment duration and disease/model background. Chao1 and Shannon reached statistical significance in the ≤4-week subgroup but not in the >4-week subgroup, whereas ACE increased in both duration strata. However, differences in within-subgroup statistical significance do not establish a true difference between treatment durations. Consistent with this, continuous treatment-duration meta-regression did not identify a significant linear moderating effect for any eligible outcome, including Chao1, Shannon, Simpson, Firmicutes, Bacteroidetes, Proteobacteria, *Bacteroides*, and *Lactobacillus*. Therefore, the observed categorical subgroup pattern cannot be interpreted as evidence that shorter treatment courses are more effective or that microbial richness necessarily responds earlier to moxibustion. The absence of a significant linear moderator also does not prove that treatment duration is biologically irrelevant. Treatment duration in days is an imperfect proxy for cumulative moxibustion exposure because treatment frequency, session duration, acupoint number, moxibustion technique, and thermal dose differed across studies. Moreover, Jia et al. observed clearer microbiota and inflammatory changes after 4 and 6 weeks than after 2 weeks, with little additional difference between 4 and 6 weeks, suggesting that non-linear or plateau responses remain possible (Jia et al., 2022). Accordingly, the meta-regression is interpreted as showing no detectable linear study-level duration-effect relationship within the available evidence, rather than as demonstrating the absence of any duration-dependent biological response.

Shannon diversity increased significantly in intestinal/digestive-related models, whereas Chao1 and ACE showed clearer increases in non-intestinal models. Bacteroidetes increased in intestinal/digestive-related models, while *Bacteroides* decreased only in the non-intestinal subgroup. *Ruminococcus*, however, did not reach statistical significance in either disease/model subgroup. These contrasting patterns support context dependence but do not establish disease category as a causal effect modifier, because disease model, treatment duration, animal species, acupoint protocol, and baseline microbial disturbance often covaried across studies.

Moxibustion may first attenuate systemic inflammatory tone and thereby allow partial microbiota recovery, or microbiota remodeling may contribute upstream to reduced inflammatory activity. A third possibility is that microbiota and inflammatory changes occur in parallel as downstream consequences of a shared host response to moxibustion, including thermal, sensory, neuroimmune, vascular, or barrier-related effects. The present meta-analysis cannot distinguish among these causal structures because microbiota and inflammatory outcomes were synthesized across studies rather than analyzed as temporally ordered mediators. Thus, the concurrence of higher α-diversity and lower TNF-α/IL-1β should be regarded as a biologically coherent association, not evidence that the microbiota changes caused the inflammatory changes. Nor does the present evidence exclude the reverse direction, in which attenuation of host inflammation creates a more permissive intestinal ecological environment and microbiota changes occur secondarily. Longitudinal and mediation-based designs are needed to distinguish these possibilities.

Only three included studies reported metabolite- or pathway-related outcomes, but these findings are relevant because microbial metabolites and functional pathways may be more directly linked to host physiology than taxonomic composition alone (Neurath et al., 2025; Zhang et al., 2024). Lin et al. reported that moxibustion increased fecal short-chain fatty acids, including acetate, propionate, and butyrate, and altered several amino acid-related metabolites (Lin et al., 2024). Short-chain fatty acids are microbial metabolites that support epithelial energy metabolism and barrier integrity and regulate mucosal and systemic immunity through mechanisms including G protein-coupled receptor signaling and histone deacetylase inhibition (Mann et al., 2024; Shen et al., 2025; Wang et al., 2024). Therefore, increased SCFAs after moxibustion are consistent with improved microbial richness and reductions in selected pro-inflammatory mediators. However, because metabolite and inflammatory outcomes were not quantitatively linked through temporal or mediation analyses, these parallel observations do not establish SCFAs as mediators of the anti-inflammatory effects of moxibustion.

In the IBD model reported by Yang et al. (2024) acupuncture and moxibustion shared some metabolic responses, whereas moxibustion additionally regulated taurine and hypotaurine metabolism, branched-chain amino acid biosynthesis, and HIF-1 signaling. Zhong et al. (2022) further reported, based on 16S rDNA-derived KEGG functional prediction, that several microbial functional pathways were reduced in Crohn’s disease model rats and partially restored after moxibustion, including pathways related to membrane transport, carbohydrate metabolism, amino acid metabolism, replication and repair, and translation. Nevertheless, metabolite evidence remains preliminary because the number of studies is small, disease backgrounds are heterogeneous, and analytical platforms differ substantially.

Taken together, the available evidence supports a hypothetical microbiota–metabolism–immunity framework. Moxibustion-related host responses may alter the intestinal ecological environment and microbial community structure; associated functional changes may in turn influence metabolites such as SCFAs, which have established roles in epithelial barrier maintenance and immune regulation, with downstream consequences for inflammatory signaling. Within this framework, *A. muciniphila* and Treg/Th17-related immune responses should be regarded as candidate mechanistic modules rather than established mediators, because neither was sufficiently supported by the present evidence. The principal mechanistic gap therefore lies in directly linking specific microbial species or strains to metabolites, barrier responses, immune-cell phenotypes, and inflammatory outcomes within the same experimental system. Future studies should integrate microbiome sequencing, untargeted and targeted metabolomics, epithelial barrier markers, and immune phenotyping within the same design to determine whether moxibustion-induced microbial shifts have reproducible functional consequences.

This review has several strengths. First, it specifically evaluated moxibustion as an independent intervention, thereby reducing the confounding that may arise when acupuncture and moxibustion are combined into a single intervention category. Second, the review followed a prospectively registered and published protocol, which improved methodological transparency, reduced the risk of *post hoc* analytical decisions (Zhang et al., 2025) and strengthened the robustness of the synthesis. Third, the analysis separated clinical and preclinical evidence, thereby improving the interpretability and translational boundary of the quantitative findings. Fourth, the use of the ratio of means for microbiota-related continuous outcomes facilitated comparison across studies reporting abundance or diversity outcomes on different numerical scales. In addition, separating outcomes with insufficient comparisons from the principal synthesis, treatment-duration meta-regression, and grading clinical and preclinical evidence separately strengthened the interpretability of the findings.

Several limitations should be acknowledged. First, the evidence base was dominated by animal studies, and the number of clinical studies was limited; therefore, direct clinical extrapolation is constrained. Although separating clinical and preclinical evidence reduced cross-species interpretational heterogeneity, substantial biological heterogeneity remained within the animal evidence because the included studies encompassed markedly different disease models. Second, substantial heterogeneity was present for many outcomes, probably reflecting differences in disease models, moxibustion protocols, acupoint combinations, treatment duration, control conditions, sequencing platforms, and analytical pipelines. Third, methodological quality was limited by incomplete reporting, particularly for allocation concealment, random housing, and blinding. Although sensitivity analyses excluding studies explicitly judged at high risk of bias did not change the statistical significance status of the principal outcomes, this does not eliminate uncertainty arising from the large number of incompletely reported domains. Fourth, formal GRADE assessment identified very-low-certainty evidence for all evaluable prespecified primary microbiota outcomes in the relevant clinical and preclinical evidence bodies. Fifth, treatment-duration meta-regression evaluated only a linear study-level relationship with calendar duration; insufficient comparable information prevented reliable analysis of cumulative moxibustion exposure, and study-level confounding and non-linear duration effects cannot be excluded. Sixth, several taxon-level outcomes and IL-17 were supported by few eligible comparisons and should therefore be regarded as hypothesis-generating rather than established effects. Seventh, only three included studies reported metabolite- or pathway-related outcomes, preventing quantitative synthesis and leaving the metabolic link between microbiota alterations and immune/inflammatory responses insufficiently established. Lastly, most of the included studies relied on 16S rRNA sequencing, which limits species- or strain-level interpretation. Consequently, the present evidence cannot determine whether higher-level taxonomic signals reflect specific organisms or strains with distinct functional properties.

## 5 Conclusion

Preclinical evidence from this systematic review and meta-analysis suggests that moxibustion is associated with increased microbial richness and diversity and reduced selected pro-inflammatory mediators, particularly TNF-α and IL-1β. Taxon-specific effects were highly heterogeneous, and the available clinical evidence was insufficient to establish corresponding effects in humans. These findings indicate the hypothesis that the gut microbiota–inflammation axis may contribute to the biological effects of moxibustion in disease states characterized by dysbiosis and inflammatory activation. However, the current evidence does not establish a causal microbiota-mediated mechanism, because most included studies are preclinical, clinical evidence is limited, and substantial heterogeneity is observed across several outcomes. Future studies should prioritize rigorously designed clinical research and mechanistic experiments integrating longitudinal microbiome profiling, metabolomics, epithelial barrier assessment, and immune phenotyping.

## Data Availability

The original contributions presented in the study are included in the article/**Supplementary Material**. Further inquiries can be directed to the corresponding author/s.
