# Supplementary material for: Moxibustion and the gut microbiota–inflammation axis: a systematic review and meta-analysis of clinical and preclinical studies
Source: Front Cell Infect Microbiol. 2026 Oct 5;16:1890021. doi: 10.3389/fcimb.2026.1890021 (PMC13677476; doi:10.3389/fcimb.2026.1890021)
Supplement: Supplementary file 1 [file DataSheet1.pdf]

Database	Search number	Query	Results
PubMed	#1	"Moxibustion"[MeSH Terms]	3011
	#2	"Moxabustion"[Title/Abstract] OR "Moxa"[Title/Abstract] OR "Mugwort"[Title/Abstract] OR "Wormwood"[Title/Abstract] OR "Artemisia"[Title/Abstract]	6967
	#3	#1 OR #2	9652
	#4	"Gastrointestinal Microbiome"[Mesh]	51223
	#5	((((((((((((((((((((((((((((((((((((Gastrointestinal Microbiome[Title/Abstract]) OR (Gastrointestinal Microbiomes[Title/Abstract])) OR (Microbiome, Gastrointestinal[Title/Abstract])) OR (Gut Microbiome[Title/Abstract])) OR (Gut Microbiomes[Title/Abstract])) OR (Microbiome, Gut[Title/Abstract])) OR (Gut Microflora[Title/Abstract])) OR (Microflora, Gut[Title/Abstract])) OR (Gut Microbiota[Title/Abstract])) OR (Gut Microbiotas[Title/Abstract])) OR (Microbiota, Gut[Title/Abstract])) OR (Gastrointestinal Flora[Title/Abstract])) OR (Flora, Gastrointestinal[Title/Abstract])) OR (Gut Flora[Title/Abstract])) OR (Flora, Gut[Title/Abstract])) OR (Gastrointestinal Microbiota[Title/Abstract])) OR (Gastrointestinal Microbiotas[Title/Abstract])) OR (Microbiota, Gastrointestinal[Title/Abstract])) OR (Gastrointestinal Microbial Community[Title/Abstract])) OR (Gastrointestinal Microbial Communities[Title/Abstract])) OR (Microbial Community, Gastrointestinal[Title/Abstract])) OR (Gastrointestinal Microflora[Title/Abstract])) OR (Microflora, Gastrointestinal[Title/Abstract])) OR (Gastric Microbiome[Title/Abstract])) OR (Gastric Microbiomes[Title/Abstract])) OR (Microbiome, Gastric[Title/Abstract])) OR (Intestinal Microbiome[Title/Abstract])) OR (Intestinal Microbiomes[Title/Abstract])) OR (Microbiome, Intestinal[Title/Abstract])) OR (Intestinal Microbiota[Title/Abstract])) OR (Intestinal Microbiotas[Title/Abstract])) OR (Microbiota, Intestinal[Title/Abstract])) OR (Intestinal Microflora[Title/Abstract])) OR (Microflora, Intestinal[Title/Abstract])) OR (Intestinal Flora[Title/Abstract])) OR (Flora, Intestinal[Title/Abstract])) OR (Enteric Bacteria[Title/Abstract])) OR (Bacteria, Enteric[Title/Abstract])	102080
	#6	#4 OR #5	110029
	#7	#3 AND #6	76
	#8	#7 AND (1000/1/1:2024/12/31[pdat])	76
Web of	#1	TS=(Moxabustion OR Moxa OR Mugwort OR Wormwood OR	25095

science		Artemisia)	
	#2	TS=(Gastrointestinal Microbiome OR Gastrointestinal Microbiomes OR Microbiome, Gastrointestinal OR Gut Microbiome OR Gut Microbiomes OR Microbiome, Gut OR Gut Microflora OR Microflora, Gut OR Gut Microbiota OR Gut Microbiotas OR Microbiota, Gut OR Gastrointestinal Flora OR Flora, Gastrointestinal OR Gut Flora OR Flora, Gut OR Gastrointestinal Microbiota OR Gastrointestinal Microbiotas OR Microbiota, Gastrointestinal OR Gastrointestinal Microbial Community OR Gastrointestinal Microbial Communities OR Microbial Community, Gastrointestinal OR Gastrointestinal Microflora OR Microflora, Gastrointestinal OR Gastric Microbiome OR Gastric Microbiomes OR Microbiome, Gastric OR Intestinal Microbiome OR Intestinal Microbiomes OR Microbiome, Intestinal OR Intestinal Microbiota OR Intestinal Microbiotas OR Microbiota, Intestinal OR Intestinal Microflora OR Microflora, Intestinal OR Intestinal Flora OR Flora, Intestinal OR Enteric Bacteria OR Bacteria, Enteric)	220687
	#3	#1 AND #2	155
		Limiters applied:<br>1. Publication Date: 2000-01-01 to 2024-12-31<br>https://webofscience.clarivate.cn/wos/alldb/summary/7052a8bc-8abf-4cb5-ae48-5bd2c1abed07-0144f33660/relevance/1	155
Cochrane Library	#1	MeSH descriptor: [Moxibustion] explode all trees	697
	#2	(Moxabustion):ti,ab,kw OR (Moxa):ti,ab,kw OR (Mugwort):ti,ab,kw OR (Wormwood):ti,ab,kw OR (Artemisia):ti,ab,kw	511
	#3	#1 OR #2	1118
	#4	MeSH descriptor: [Gastrointestinal Microbiome] explode all trees	1898
	#5	(Gastrointestinal Microbiome):ti,ab,kw OR (Gastrointestinal Microbiomes):ti,ab,kw OR (Microbiome, Gastrointestinal):ti,ab,kw OR (Gut Microbiome):ti,ab,kw OR (Gut Microbiomes):ti,ab,kw	4582
	#6	(Microbiome, Gut):ti,ab,kw OR (Gut Microflora):ti,ab,kw OR (Microflora, Gut):ti,ab,kw OR (Gut Microbiota):ti,ab,kw OR (Gut Microbiotas):ti,ab,kw	7439
	#7	(Microbiota, Gut):ti,ab,kw OR (Gastrointestinal Flora):ti,ab,kw OR (Flora, Gastrointestinal):ti,ab,kw OR (Gut Flora):ti,ab,kw OR (Flora, Gut):ti,ab,kw	6777
	#8	(Gastrointestinal Microbiota):ti,ab,kw OR (Gastrointestinal Microbiotas):ti,ab,kw OR (Microbiota, Gastrointestinal):ti,ab,kw OR (Gastrointestinal Microbial Community):ti,ab,kw OR (Gastrointestinal Microbial Communities):ti,ab,kw	3101
	#9	(Microbial Community, Gastrointestinal):ti,ab,kw OR (Gastrointestinal Microflora):ti,ab,kw OR (Microflora, Gastrointestinal):ti,ab,kw OR (Gastric Microbiome):ti,ab,kw OR (Gastric Microbiomes):ti,ab,kw	1084

	#10	(Microbiome, Gastric):ti,ab,kw OR (Intestinal Microbiome):ti,ab,kw OR (Intestinal Microbiomes):ti,ab,kw OR (Microbiome, Intestinal):ti,ab,kw OR (Intestinal Microbiota):ti,ab,kw	3498
	#11	(Intestinal Microbiotas):ti,ab,kw OR (Microbiota, Intestinal):ti,ab,kw OR (Intestinal Microflora):ti,ab,kw OR (Microflora, Intestinal):ti,ab,kw OR (Intestinal Flora):ti,ab,kw	4623
	#12	(Flora, Intestinal):ti,ab,kw OR (Enteric Bacteria):ti,ab,kw OR (Bacteria, Enteric):ti,ab,kw	2832
	#13	#4 OR #5 OR #6 OR #7 OR #8 OR #9 OR #10 OR #11 OR #12	11726
	#14	#3 AND #13	8
CNKI		(SU%'艾灸' + '灸法' + '灸') and (SU%'肠道微生物' + '肠道菌群' + '肠道微生态')	303
		时间范围：至 2024-12-31	301
Wanfang Data		主题= (艾灸 OR 灸法 OR 灸) AND 主题= (肠道微生物 OR 肠道菌群 OR 肠道微生态)	495
		发表时间：不限-2024 年	495
VIP		U=(艾灸 OR 灸法 OR 灸) AND U=(肠道微生物 OR 肠道菌群 OR 肠道微生态)	292
		时间范围：2000-01 至 2024-12	292
