# Supplementary material for: Moxibustion and the gut microbiota–inflammation axis: a systematic review and meta-analysis of clinical and preclinical studies
Source: Front Cell Infect Microbiol. 2026 Oct 5;16:1890021. doi: 10.3389/fcimb.2026.1890021 (PMC13677476; doi:10.3389/fcimb.2026.1890021)
Supplement: Supplementary file 2 [file DataSheet2.pdf]

## Supplementary Table S2. Outcomes with limited preclinical evidence

| Category | Outcome | k | Effect measure | Pooled effect (95% CI) | P | $I^2$ (%) |
|---|---|---|---|---|---|---|
| Phylum | Verrucomicrobia | 4 | ROM | 9.567 (2.103 to 43.519) | 0.0035 | 98.7 |
| Genus/family | *Bifidobacterium* | 4 | ROM | 1.183 (0.436 to 3.215) | 0.7414 | 99.4 |
| | Enterobacteriaceae | 2 | ROM | 3.410 (2.575 to 4.516) | <0.0001 | 0.0 |
| | *Escherichia* | 4 | ROM | 0.408 (0.112 to 1.489) | 0.1747 | 98.9 |
| | *Roseburia* | 3 | ROM | 1.053 (0.331 to 3.349) | 0.9302 | 98.9 |
| Inflammatory mediators | IL-17 | 3 | SMD | -0.478 (-1.115 to 0.158) | 0.1407 | 0.0 |

Note: For microbiota outcomes, $k < 5$ was below the prespecified quantitative-synthesis threshold.

ROM, ratio of means; SMD, standardized mean difference; CI, confidence interval; $I^2$, heterogeneity statistic.
