# Supplementary material for: Moxibustion and the gut microbiota–inflammation axis: a systematic review and meta-analysis of clinical and preclinical studies
Source: Front Cell Infect Microbiol. 2026 Oct 5;16:1890021. doi: 10.3389/fcimb.2026.1890021 (PMC13677476; doi:10.3389/fcimb.2026.1890021)
Supplement: Supplementary file 3 [file DataSheet3.pdf]

Supplementary Table S3. Sensitivity analysis excluding explicit high-risk-of-bias animal studies

Domain	Outcome	Main analysis	High-RoB study/studies removed	Sensitivity pooled effect
Alpha diversity	Chao1	ROM 1.079 [1.034 to 1.127]; P = 0.0005; I^2 = 86.8%	Fan 2023, Wang 2022	ROM 1.071 [1.022 to 1.122]; P = 0.0040; I^2 = 87.8%
	ACE	ROM 1.100 [1.044 to 1.160]; P = 0.0004; I^2 = 73.0%	Wang 2022	ROM 1.101 [1.042 to 1.164]; P = 0.0007; I^2 = 75.9%
	Shannon	ROM 1.047 [1.015 to 1.080]; P = 0.0037; I^2 = 80.7%	Fan 2023, He 2023, Wang 2022	ROM 1.044 [1.009 to 1.080]; P = 0.0128; I^2 = 77.7%
	Simpson	ROM 1.004 [0.990 to 1.019]; P = 0.5685; I^2 = 80.8%	Wang 2022	ROM 1.011 [0.999 to 1.023]; P = 0.0639; I^2 = 67.2%
Phylum	Firmicutes	ROM 0.954 [0.843 to 1.080]; P = 0.4608; I^2 = 95.6%	He 2023, Wang 2022	ROM 0.961 [0.841 to 1.097]; P = 0.5540; I^2 = 96.1%
	Bacteroidetes	ROM 0.956 [0.795 to 1.149]; P = 0.6292; I^2 = 96.3%	He 2023, Wang 2022	ROM 0.953 [0.766 to 1.187]; P = 0.6681; I^2 = 96.6%
	Proteobacteria	ROM 0.996 [0.634 to 1.563]; P = 0.9847; I^2 = 98.2%	He 2023, Wang 2022	ROM 0.858 [0.536 to 1.374]; P = 0.5245; I^2 = 98.2%
	Actinobacteria	ROM 0.786 [0.518 to 1.194]; P = 0.2597; I^2 = 97.3%	None	ROM 0.786 [0.518 to 1.194]; P = 0.2597; I^2 = 97.3%
Genus/family	*Bacteroides*	ROM 0.715 [0.476 to 1.073]; P = 0.1049; I^2 = 98.1%	Fan 2023, He 2023, Wang 2022	ROM 0.701 [0.427 to 1.150]; P = 0.1598; I^2 = 98.2%
	Lactobacillus	ROM 0.913 [0.658 to 1.268]; P = 0.5872; I^2 = 98.6%	Bao 2019, He 2023, Wang 2022	ROM 0.852 [0.522 to 1.392]; P = 0.5229; I^2 = 98.5%
	Lachnospiraceae	ROM 0.785 [0.466 to 1.321]; P = 0.3618; I^2 = 96.2%	Fan 2023, Wang 2022	ROM 0.935 [0.511 to 1.710]; P = 0.8269; I^2 = 96.5%
	Prevotella	ROM 0.761 [0.520 to 1.114]; P = 0.1604; I^2 = 95.3%	He 2023	ROM 0.712 [0.470 to 1.080]; P = 0.1103; I^2 = 95.0%
	Ruminococcus	ROM 0.643 [0.426 to 0.972]; P = 0.0364; I^2 = 96.9%	Fan 2023, He 2023	ROM 0.578 [0.348 to 0.960]; P = 0.0343; I^2 = 97.5%
Inflammatory marker	TNF-α	SMD -1.648 [-2.659 to -0.637]; P = 0.0014; I^2 = 82.5%	None	SMD -1.648 [-2.659 to -0.637]; P = 0.0014; I^2 = 82.5%
	IL-6	SMD -0.665 [-1.448 to 0.118]; P = 0.0961; I^2 = 68.0%	None	SMD -0.665 [-1.448 to 0.118]; P = 0.0961; I^2 = 68.0%
	IL-1β	SMD -1.861 [-2.419 to -1.303]; P < 0.0001; I^2 = 0.0%	Bao 2019	SMD -1.691 [-2.323 to -1.059]; P <0.0001; I^2 = 0.0%
	IL-10	SMD 0.439 [-0.657 to 1.534]; P = 0.433; I^2 = 84.3%	None	SMD 0.439 [-0.657 to 1.534]; P = 0.4326; I^2 = 84.3%

Note: Sensitivity analysis excludes animal studies with an explicit High-RoB judgement: He 2023, Fan 2023, Wang 2022, and Bao 2019 where applicable. ROM, ratio of means; SMD, standardized mean difference; CI, confidence interval.
