# Supplementary material for: Moxibustion and the gut microbiota–inflammation axis: a systematic review and meta-analysis of clinical and preclinical studies
Source: Front Cell Infect Microbiol. 2026 Oct 5;16:1890021. doi: 10.3389/fcimb.2026.1890021 (PMC13677476; doi:10.3389/fcimb.2026.1890021)
Supplement: Supplementary file 4 [file DataSheet4.pdf]

Supplementary Table S4. Continuous treatment-duration meta-regression

Category	Outcome	k	β per day	95% CI for β	P for moderator	ROM ratio per +7 days (95% CI)	Residual τ^2	R^2 analogue (%)
α- diversity	Chao1	17	-0.0011	-0.0026 to 0.0003	0.1316	0.9921 (0.9820 to 1.0024)	0.0087	0
	Shannon	18	-0.0002	-0.0012 to 0.0008	0.7064	0.9986 (0.9916 to 1.0057)	0.0047	0
	Simpson	11	0.0001	-0.0009 to 0.0010	0.9117	1.0004 (0.9939 to 1.0069)	0.0009	0
Phylum	Firmicutes	17	0.0015	-0.0025 to 0.0056	0.4551	1.0108 (0.9827 to 1.0398)	0.0850	0
	Bacteroidetes	17	0.0021	-0.0034 to 0.0076	0.4549	1.0148 (0.9764 to 1.0547)	0.1518	0
	Proteobacteria	15	-0.0019	-0.0151 to 0.0113	0.7761	0.9867 (0.8997 to 1.0821)	0.9220	0
Genus/family	*Bacteroides*	17	0.0018	-0.0095 to 0.0130	0.7585	1.0124 (0.9359 to 1.0951)	0.7338	0
	Lactobacillus	18	-0.0043	-0.0164 to 0.0078	0.4872	0.9704 (0.8916 to 1.0562)	0.8213	0

Note: Univariable random-effects meta-regression used treatment duration in days as a continuous study-level moderator for outcomes with at least 10 eligible comparisons and sufficient independent study-level information. β represents the change in log(ROM) per additional treatment day; the ROM ratio per +7 days is provided for interpretability.
