# Supplementary material for: Moxibustion and the gut microbiota–inflammation axis: a systematic review and meta-analysis of clinical and preclinical studies
Source: Front Cell Infect Microbiol. 2026 Oct 5;16:1890021. doi: 10.3389/fcimb.2026.1890021 (PMC13677476; doi:10.3389/fcimb.2026.1890021)
Supplement: Supplementary file 5 [file DataSheet5.pdf]

Supplementary Table S5. Small-study effects for prespecified animal primary outcomes

Outcome	Animal-only k	Egger intercept	SE	t	df	P
Shannon	21	-1.0107	0.8892	-1.1366	19	0.2698
Simpson	12	-1.8801	0.6189	-3.0376	10	0.0125
Firmicutes	19	-0.5304	1.9128	-0.2773	17	0.7849
Bacteroidetes	19	-2.3852	2.5226	-0.9455	17	0.3576
Lactobacillus	21	-1.4152	2.5093	-0.5640	19	0.5793
