# Supplementary material for: Moxibustion and the gut microbiota–inflammation axis: a systematic review and meta-analysis of clinical and preclinical studies
Source: Front Cell Infect Microbiol. 2026 Oct 5;16:1890021. doi: 10.3389/fcimb.2026.1890021 (PMC13677476; doi:10.3389/fcimb.2026.1890021)
Supplement: Supplementary file 6 [file DataSheet6.pdf]

Supplementary Table S6. GRADE Summary of Findings for primary microbiota outcomes

Evidence body	Outcome	Evidence base	Effect / direction	Risk of bias	Inconsistency	Indirectness	Imprecision	Publication bias	Certainty	Reason for downgrading
Preclinical	Shannon	21 comparisons	ROM 1.047 [1.015 to 1.080]; I^2 = 80.7%	Serious (-1)	Serious (-1)	Serious (-1)	Not serious	Not serious	Very low ⊕◯◯◯	Pervasive unclear reporting across animal RoB domains, considerable unexplained heterogeneity, and indirectness from diverse animal models/species to human clinical effects.
Preclinical	Simpson	12 comparisons	ROM 1.004 [0.990 to 1.019]; I^2 = 80.8%	Serious (-1)	Serious (-1)	Serious (-1)	Serious (-1)	Serious (-1)	Very low ⊕◯◯◯	Considerable heterogeneity, CI crosses no effect, and evidence of funnel-plot asymmetry/small-study effects in addition to animal-to-human indirectness.
Preclinical	Firmicutes	19 comparisons	ROM 0.954 [0.843 to 1.080]; I^2 = 95.6%	Serious (-1)	Very serious (-2)	Serious (-1)	Serious (-1)	Not serious	Very low ⊕◯◯◯	Extremely heterogeneous effects across models and protocols; CI includes potentially meaningful decreases and increases; translatability to human effects is limited.
Preclinical	Bacteroidetes	19 comparisons	ROM 0.956 [0.795 to 1.149]; I^2 = 96.3%	Serious (-1)	Very serious (-2)	Serious (-1)	Serious (-1)	Not serious	Very low ⊕◯◯◯	Extremely heterogeneous effects with a wide CI crossing no effect, plus substantial biological/model indirectness.
Preclinical	*Lactobacillus*	21 comparisons	ROM 0.913 [0.658 to 1.268]; I^2 = 98.6%	Serious (-1)	Very serious (-2)	Serious (-1)	Serious (-1)	Not serious	Very low ⊕◯◯◯	Very high unexplained heterogeneity and wide CI compatible; diverse models and microbiota methods limit translation.
Preclinical	*Bifidobacterium*	4 comparisons	ROM 1.183 [0.436 to 3.215]; I^2 = 99.4%	Serious (-1)	Very serious (-2)	Serious (-1)	Very serious (-2)	Not assessed; k<10	Very low ⊕◯◯◯	Very sparse and extremely inconsistent animal evidence, with a very wide interval and strong indirectness.
Clinical	Firmicutes	3 RCTs; n = 207 analyzed	Study-specific ROMs: 0.935, 1.068, 1.053; all CIs include or nearly include no effect	Serious (-1)	Serious (-1)	Not serious	Serious (-1)	Not assessed; k<10	Very low ⊕◯◯◯	One contributing trial is high risk overall and the others have unclear domains; study estimates are not consistent enough for a stable direction and precision is insufficient for a reliable pooled clinical effect.
Clinical	Bacteroidetes	3 RCTs; n = 207 analyzed	Study-specific ROMs: 0.981, 1.053, 0.909	Serious (-1)	Serious (-1)	Not serious	Serious (-1)	Not assessed; k<10	Very low ⊕◯◯◯	Direction varies across studies and the evidence is too sparse for a precise pooled clinical estimate; risk-of-bias concerns remain.
Clinical	*Lactobacillus*	3 RCTs; n = 438 analyzed	Chen 1.084↑; An 1.209↑; Wang CD 0.758↓	Serious (-1)	Very serious (-2)	Not serious	Not serious	Not assessed; k<10	Very low ⊕◯◯◯	Two trials show increases whereas one shows a clear decrease; assay methods and clinical populations differ, producing major inconsistency despite relatively precise individual estimates.
Clinical	*Bifidobacterium*	5 RCTs; n = 555 analyzed	Chen 1.090↑; An 1.208↑; Wang CD 1.120 (CI crosses 1); Jing 0.368↓; Lin: non-significant increase	Serious (-1)	Very serious (-2)	Not serious	Not serious	Not assessed; k<10	Very low ⊕◯◯◯	Clinical findings are directionally contradictory, with a large decrease in Jing 2021 versus increases/tendencies in the other studies; one contributing study is high risk overall and quantitative pooling remains below the prespecified threshold.

Note: GRADE was applied separately by outcome and evidence body. "Not assessed" indicates that formal small-study-effect testing was not performed because fewer than 10 eligible comparisons were available. No eligible clinical Shannon or Simpson data were available; these outcomes were therefore not graded.
